# Model-based estimation of the economic burden of cholera in Africa

**DOI:** 10.1136/bmjopen-2020-044615

**Published:** 2021-03-23

**Authors:** Vittal Mogasale, Samuel Mwaura Ngogoyo, Vijayalaxmi V Mogasale

**Affiliations:** 1 Policy and Economic Research, International Vaccine Institute, Gwanak-gu, Korea; 2 Department of Health, County government of Nyandarua, Ol Kalou, Kenya; 3 Department of Pediatrics, Yenepoya Medical College Hospital, Mangalore, India

**Keywords:** health economics, health policy, public health, tropical medicine

## Abstract

**Objectives:**

To estimate the economic burden of cholera in Africa.

**Settings:**

Cholera affected 44 countries in Africa.

**Participants:**

The analysis used data from public sources in Africa published until September 2019.

**Methods:**

Based on existing data from field-based cost-of-illness studies, estimated cholera incidence rates, and reported cholera cases to WHO, this research estimates the economic burden of cholera in Africa from a societal perspective with 2015 as the base year. The estimate included out-of-pocket costs, public health system costs, productivity loss related to illness and an optional productivity loss related to premature deaths valued by the human capital approach. As various input data such as cholera incidence, hospitalisation rates and the number of workdays lost were not well defined, a series of scenario analyses and uncertainty analyses, accounting for unknowns and data variability, was conducted. Similarly, the value of time lost due to illness and deaths using the human capital approach was explored through scenario analyses.

**Results:**

In 2015, an estimated 1 008 642 cases in 44 African countries resulted in an economic burden of US$130 million from cholera-related illness and its treatment. When the estimated 38 104 cholera deaths were included in the analysis, the economic burden increased to US$1 billion or international $2.4 billion for the same year. At the same time, when only the 71 126 cases and 937 deaths reported to the WHO are considered, the economic burden was only US$68 million for the year 2015. The estimates of economic burden are thus heavily dependent on the cholera incidence rate, how time lost due to illness and deaths are calculated, hospitalisation rates and hospitalisation costs.

**Conclusion:**

The findings can be used as an economic justification for cholera control in Africa and for generating value-for-money evidence to underpin Ending Cholera—A Global Roadmap to 2030 with considerations to study limitations.

**Strengths and limitations of this study**

- Model-based estimation of the economic burden of cholera in 44 African countries was conducted using various secondary data sources.
- The average inpatient health system costs, out-of-pocket expenses and the number of days of productivity lost related to cholera treatment from five African papers that presented data was directly applied to countries that did not have data.
- The estimated number of cholera cases was derived from the global burden of disease study and WHO report.
- The productivity loss related to cholera illness and premature deaths were valued by the human capital approach using gross domestic product per capita.
- Cholera incidence rates, cholera hospitalisation rates and cholera hospitalisation costs are the most uncertain parameters that drove the results.

## Introduction

Cholera, an acute, diarrhoeal illness caused by infection of the intestine with the toxigenic bacterium *Vibrio cholerae* is linked to poor water, sanitation and hygiene (WASH) conditions.^1^ In cholera-endemic countries, an estimated 2.8 million cases and 91 000 deaths occur annually, due to cholera.^2^ However, the actual number of reported cases to WHO is significantly lower. Globally, around 132 000 cases and 2400 deaths were reported to the WHO in 2016.^3^ Cumulatively, from 2000 to 2016, 3.4 million cholera cases and 65 000 deaths worldwide were reported to WHO.^3^ Countries in Africa continue to experience a disproportionately high number of cholera outbreaks resulting in a high burden of disease and deaths. In 2016, 17 countries in Africa accounted for 71 000 cases (54% of global cases) with 1760 deaths (42%of deaths globally).^3^ Furthermore, the average case fatality rate of 2.5% in African countries is substantially higher than the global average of 1.8%.^3^

When measured as an economic burden, cholera has a significant economic impact on health systems and individuals. The societal cost per cholera case is believed to be more than US$1000 when the wider socioeconomic costs such as cost to the health system, the patient’s family and income loss due to death, are taken into account.^4^ While several studies have been done in various countries on the economic burden of cholera, the cost-effectiveness of interventions and other related areas of interest, there is a lack of any recent studies estimating the economic burden in Africa looking into the areas of loss of productivity, hospitalisation and outpatient costs, quantified at the national and regional levels. A model-based estimate in 2007 reported an annual economic loss ranging from US$39 million to US$64.2 million as a result of cholera in US$2002 prices and based on regional life expectancies ranging from 40 to 73 years in Africa.^5^ The study used several cost assumptions around standard treatment and diagnostic practices which may not be the current situation in Africa and may be outdated.

A recent economic burden estimate in Asia used the actual estimated cost of illness collected from countries to project the economic burden while using a variety of scenario and sensitivity analyses to demonstrate the uncertainties around the estimated and reported cholera cases to WHO.^6^ This kind of economic burden estimate provides a better understanding of economic offsets that can be achieved through cholera control measures and can be used as evidence to make a case for the effectiveness of cholera control. We used the same methodological approach as in Asia^6^ and used the latest data available from field settings in Africa to quantify the economic burden of cholera in Africa.

## Methods

The research involved the selection of countries, identification of cost-of-illness studies to define out-of-pocket (OOP) expenses, productivity loss and health system costs to estimate and extrapolate the economic burden, and finally, conduct sensitivity analysis and scenario analyses. Patients or the public were not involved in the design, or conduct, or reporting, or dissemination plans of this research.

### Conceptual framework

The primary input needed for estimating the economic burden is the cost-of-illness studies conducted around cholera cases at health facilities in African countries. Such studies provide three sets of key information.^1^ OOP expenditures borne by patients and families for cholera treatment which could include medical costs such as medicines and intravenous fluids, or non-medical costs expenditures on transport and food.^2^ Loss of income, which is often referred to as productivity costs related to illness, because the patient is unable to work and someone needs to take care of the patient. This is often presented in the number of workdays lost.^3^ Cost to the health system for providing the cholera treatment which includes costs related to staff, provision of services, maintenance of hospital facilities.

The cost-of-illness data are typically not available from most African countries. In such situations, cost-of-illness was extrapolated to African countries using available data from field studies in the region (OOP expenses, inpatients costs, and productivity loss) and WHO estimates (outpatient costs) such that each country has a unit cost of illness (cost of illness per cholera case). When the total number of cholera cases is multiplied by the unit cost-of-illness, the economic burden for that country is derived which is summated at the regional level. However, all the input costs and the number of cases are not constant numbers. There may be several biases and reported variations such as under-reporting or overestimations because of which we conduct a series of scenario analyses and uncertainty analyses as described below so that the limitations and boundaries are well understood.

### Country selection and disease burden estimation

African countries as defined by the United Nations,^7^ that either reported cholera cases to WHO or were estimated to have cholera cases, were selected for the analysis. The year 2015 was selected as the reference year for data comparability purposes since both, disease burden estimates^2^ and cases reported to WHO,^8^ were available for that year. Most often than not, cholera is under-reported by many countries because of reasons such as the lack of diagnostics and reagents to test cholera, inadequacies in the surveillance system, differences in case definition, and the reluctance of authorities to acknowledge or report cholera.^9^ Therefore, the data in the WHO report is under-reported and an economic burden estimate based on the WHO report will be an underestimation. Whereas the 2015 global burden of disease estimate^2^ has considered several incidence scenarios as part of sensitivity analysis, of which three were used in this analysis. In total, four cholera incidence scenarios were used in the analysis as listed below.

1. Base case: The Global Burden of Cholera study^2^ had extrapolated the cholera incidence data from Beira, Mozambique^10^ to the rest of Africa after applying the incidence rate to the population at risk based on the percentage of people without sustainable access to improved water. We used the same incidence numbers in the base case. This methodology assumes that only a part of the population is at risk for cholera. This was one of the sensitivity analyses in the Global Burden of Cholera study.^2^
2. Liberal case: The incidence rate from Beira, Mozambique was extrapolated to the rest of Africa after applying an incidence correction assumption by African WHO mortality strata D (AFR-D).^11^ It was assumed that AFR-D had half the incidence as Beira, while the rest of African countries were assumed to have the same incidence rate as Beira.^2^ Here, the assumption is that the whole of the population is at risk for cholera, but with different incidence rates. This was the base case in the Global Burden of Cholera publication.^2^
3. Conservative case: As other sensitivity analyses used in the 2015 global burden of disease estimate produced a similar number of cholera cases in Africa, a conservative estimate was derived by applying half of the incidence rate as the base case.
4. WHO report case: A separate scenario analysis was conducted based on the number of cholera cases and deaths reported to WHO in 2015.

The 2015 global burden of disease study^2^ had applied a 3.8% case fatality rate to estimate death, and the same was applied in the first three scenario analyses.

### Cholera cost-of-illness

As described previously in the conceptual framework, there are three types of costs: OOP costs, productivity loss and health system costs. These costs were extracted from literature based on actual studies conducted in healthcare settings. A previous systematic literature review^4^ had identified two cost-of-illness studies conducted in Africa, one in Mozambique^12^ and another in Tanzania.^13^ Furthermore, a PubMed search in September 2019 yielded three more studies published subsequently—one each from Malawi,^14^ Ghana^15^ and Zambia.^16^ Data from these five studies were extracted and used as inputs in the analysis.

#### OOP costs

The OOP costs for hospitalised cases were reported for all five papers used in the study, while only Malawi reported the OOP costs for outpatient cases. There are two types of OOP costs, direct medical and direct non-medical costs. Direct medical costs include expenditure accrued by patients for diagnosis, medicines and other costs directly related to the treatment of cholera. Direct non-medical costs include expenditures on travel to the healthcare facility, room and boarding, food, and other costs not directly related to treatment. All the costs reported in the different years were converted to 2015 US dollars by adjusting them based on annual percent changes in world consumer prices as reported by the International Monetary Fund (IMF).^17^ For countries that did not report OOP costs either for hospitalisation or outpatient, the average cost derived from the rest of the reported costs were applied directly.

#### Lost productivity

Loss of productivity due to illness was estimated based on the average number of lost workdays for patients and caregivers throughout the illness and the recuperation period documented separately for patients and caregivers. Lost workdays for Ghana, Malawi and Mozambique were extracted directly from their respective sources.^12 14 15^ Data from Tanzania only include the duration of illness and does not account for the loss of productivity during the recuperation period.^13^ While the study in Zambia did not give an exact average, we were able to estimate the average based on the survey results.^16^ The ranges of the lost days were calculated based on the range derived from the study in Tanzania, where the variability of 20% was observed. The lost days were then multiplied by the average gross domestic productivity (GDP) per capita^18^ to derive total productivity loss per cholera episode. As cholera incidence may be higher in people with low income, we used three scenarios that estimated productivity loss at 75%, 50% and 25% of GDP per capita.

#### Health system costs

Service delivery costs were also extracted from the papers for each hospitalised and outpatient cases. Health facility costs for service delivery included personnel, medicines, diagnostic tools, medical equipment, infrastructure, beds and utilities that patients are not responsible for paying. Of the five publications used in this study, Malawi, Tanzania and Mozambique reported health service delivery costs for hospitalised cases. For countries without reported costs, the mean hospitalisation health facility costs for these three African countries were applied. Only Malawi reported outpatient service delivery costs. The country-specific average outpatient costs for health centres with and without beds from the WHO-CHOICE^19^ project were applied to all other countries.

#### Loss of productivity because of death

To estimate the productivity loss due to premature death from cholera, the mean age of cholera death was derived from a multicountry cholera surveillance project that reported 873 deaths in six African surveillance zones.^20^ Life expectancy at birth for each country was subsequently subtracted by the mean age of cholera. Information for life expectancy used the 2015 data provided by the World Bank.^21^ To convert the years lost to monetary values, GDP per capita^18^ was multiplied by the number of deaths from cholera, and the number of productive years lost per death. A discount rate of 3% was applied to future earning potential. The GDP per capita although may be considered an overestimate considering cholera may disproportionately affect poor people, it allows accounting for everyone affected equally and their variable life expectancy.

#### Hospitalisation rate

Only the study conducted in Malawi reported a hospitalisation rate, at 90% hospitalised and 10% receiving care for less than 12 hours, which is analogous to outpatient care. Other studies included in the analysis were unclear whether all cases were hospitalised, or only hospitalised cases were accounted for. Since 90%–100% hospitalisation is considered high, the analysis was conducted based on three scenarios: (1) under the assumption of 90% hospitalisation, (2) average of 75% hospitalisation and (3) average of 55% hospitalisation. Assumption (2) was determined based on a previous investment case study^22 23^ while assumption (3) was based on results from an economic burden study conducted for Asian countries^6^ which is the average hospitalisation rate in Asia.

#### Probabilistic multivariate sensitivity analysis

A probabilistic multivariate sensitivity analysis was conducted using Ersatz.^24^ A beta-PERT distribution was used for cost inputs which use the minimum, mean and maximum as input parameters. The beta-PERT distribution is most suited for cost modelling or parameters obtained through the survey that present minimum, a maximum and most likely values.^24^ To estimate 95% CIs a Monte Carlo simulation was conducted based on 5000 random draws for each input parameter based on the distribution allocated.

#### Scenario analysis

Among all input parameters, disease burden (the number of cases) and hospitalisation rates had limited information that could be captured in one single number with uncertainty ranges. Therefore, scenario analyses were conducted for both disease burden and hospitalisation rates. Disease burden scenarios included three estimates as explained earlier and is based on a previous publication^2^ and the reported cholera cases and deaths to WHO.^8^ The hospitalisation rate had three scenarios as described above namely 90%, 75% and 55% hospitalisation rates. As productivity loss is driving overall costs, we conducted scenario analysis by valuing loss of workdays at 75%, 50% and 25% of GDP per capita.

All the costs were converted to their equivalent value in 2015 US dollars (US$). For the conversion, the annual percent changes in world consumer prices as established by the IMF were applied.^17^ The results were presented both in 2019 US$ and 2019 International dollars (I$). For the conversion from US$ to I$, a country-specific multiplication factor from World Bank data that ranged from 1.63 to 3.67 was used.^18^

#### The comparison of methodology with previous economic burden study

The previous cholera economic burden study estimated the costs in 2002 prices for WHO reported cholera cases in 2005, 2006 and 2007.^5^ It used WHO data sources and assumptions based on standard clinical practices for estimating health system (hotel cost, diagnostic costs and medicine costs) and OOP (household) costs. A methodological comparison table is provided in online supplemental annex 1. The current study estimated cholera economic burden in Africa using available data from the field-based cost of illness study and most recent disease burden estimates available and in a comparable approach to economic burden estimate in Asia.^6^

10.1136/bmjopen-2020-044615.supp1 Supplementary data

## Results

Based on a literature review, average workday loss of 5.76 (range 2.96–9.60) and 3.86 (range 3.09–4.64) was estimated for cholera cases and caregivers, respectively, irrespective of their hospitalisation status (table 1). The OOP costs were US$ 38.87 (range US$6.07–US$109.94) for hospitalised cases and US$ 5.71 (range US$0.89–US$16.15) for those who visited the health facility but did not get hospitalised. The health facility costs were estimated at US$ 65.77 and US$ 2.84 for hospitalised and outpatient cases, respectively. The mean age of death due to cholera was 23.74 years. The analysis included 44 African countries and the list of countries included in the analysis is available in online supplemental annex 2.

**Table 1:** Input parameter assumptions used in uncertainty analysis

Input parameter	Mean value	Minimum value	Maximum value	Source
Out-of-pocket costs to patient and family for hospitalisation	US$38.87	US$6.05	US$109.94	12–16
Out-of-pocket costs to patient and family for outpatient cases	US$5.71	US$0.89	US$16.15	14
No of days with the loss of income—cholera cases	5.76 days	4.61 days	6.97 days	12–16
No of days with the loss of income—caregivers	3.86 days	3.09 days	4.64 days	12 14 15
Public health service delivery costs for hospitalised cases	US$65.77	US$39.83	US$85.41	12–14
Public health service delivery costs for outpatient cases	US$2.84	US$2.55	US$3.31	14
Age of death due to cholera (years)	23.74	1.00	66.00	20
Proportion of cases hospitalised				
Base case	0.90	NA	NA	12 14 15
Scenario 1	0.75	NA	NA	22 23
Scenario 2	0.55	NA	NA	6

NA, not available.

In the base-case analysis, 1 008 642 cases of all ages were included from 44 African countries, while 71 176 cholera cases from 16 countries were reported to WHO (online supplemental annex 2). The number of deaths estimated in the base case was 38 104 from 44 countries, while 937 were reported to WHO. Excluding deaths, the total economic burden is US$130 million and I$ 315 million for the year 2015 (table 2). When productivity losses related to deaths are taken into account, the total economic burden increases to US$1 billion and I$2.4 billion for the same year. Country-specific economic burden and unit costs are provided in online supplemental annex 2, which shows the per capita economic burden of cholera ranges from US$0.1 to US$3.9 with an average of US$1.1.

**Table 2:** Economic burden of cholera in Africa

Economic burden	US$ 2015 (in millions)			I$ 2015 (in millions)		
	Mean	95% LCI	95% UCI	Mean	95% LCI	95% UCI
Lost productivity due to illness	$30.7	$22.4	$ 39.1	$72.8	$53.7	$ 92.1
Public health system costs	$58.9	$ 46.4	$70.8	$145.0	$114.8	$173.4
Out-of-pocket costs	$40.1	$16.2	$69.6	$97.6	$40.0	$168.4
Total economic burden excluding deaths	$129.6	$100.3	$164.2	$315.3	$245.0	$397.8
Lost productivity due to premature deaths	$876.2	$293.6	$1174.8	$2091.1	$721.7	$2793.0
Total economic burden including deaths	$1005.8	$426.4	$1304.1	$2406.4	$1043.9	$3107.5

LCI, Lower Confidence Interval; UCI, Upper Confidence Interval.

### Scenario analyses

The scenario analyses showed that when assumptions on the number of cholera cases changed, the economic burden excluding death ranged from US$64.6 million to US$227.2 million. The estimated/reported number of cases influences the economic burden considerably and drops to US$8.6 million when WHO reported cholera cases are considered in the analysis (table 3).

**Table 3:** Economic burden of cholera in Africa under various cholera incidence scenarios

	US$ 2015				I$ 2015			
	Base estimate	Sensitivity 1	Sensitivity 2	WHO report	Base estimate	Sensitivity 1	Sensitivity 2	WHO report
Cases	1 008 642	1 756 703	504 321	71 176	1 008 642	1 756 703	504 321	71 176
Deaths	38 104	66 416	19 052	937	38 104	66 416	19 052	937
Productivity loss due to illness (millions)	$30.7	$53.9	$15.2	$1.7	$72.8	$131.8	$36.1	$4.0
Public health system costs (millions)	$58.9	$103.0	$29.4	$4.1	$145.0	$260.0	$72.5	$9.5
Out-of-pocket costs (millions)	$40.1	$70.3	$20.0	$2.8	$97.6	$176.2	$49.6	$6.2
Total economic burden excluding deaths (millions)	$129.6	$227.2	$64.6	$8.6	$315.3	$568.0	$157.2	$19.7
Productivity loss due to premature deaths (millions)	$876.2	$1600.1	$454.7	$20.8	$2091.1	$3925.1	$1084.6	$48.1
Total economic burden including deaths (millions)	$1005.8	$1827.3	$519.3	$29.4	$2406.4	$4493.1	$1241.8	$67.8

The scenario analysis that varied hospitalisation rates had a relatively small impact on the overall economic burden. When workdays lost are valued at 75%, 50% and 25% of GDP per capita, a large reduction of economic burden was observed (table 4).

**Table 4:** Economic burden of cholera in Africa under various hospitalisation and GDP per capita scenarios

	Bases case	Hospitalisation rate 1*	Hospitalisation rate 2*	GDP 1†	GDP 2†	GDP 3†
Cases	1 008 642	1 008 642	1 008 642	1 008 642	1 008 642	1 008 642
Deaths	38 104	38 104	38 104	38 104	38 104	38 104
Hospitalisation rate (%)	90	75	55	90	90	90
Value of workdays as percentage of GDP per capita per day (%)	100	100	100	75	50	25
Productivity loss due to illness (millions US$)	$30.5	$30.5	$30.5	$22.9	$15.3	$7.6
Public health system costs (millions US$)	$58.9	$49.9	$38.0	$58.9	$58.9	$58.9
Out-of-pocket costs (millions US$)	$39.9	$34.7	$27.7	$39.9	$39.9	$39.9
Subtotal economic burden (millions US$)	$129.2	$115.1	$96.2	$121.6	$114.0	$106.3
Productivity loss due to premature deaths (millions US$)	$909.3	$909.3	$909.3	$682.0	$454.7	$227.3
Total economic burden (millions US$)	$1038.6	$1024.4	$1005.6	$803.6	$568.6	$333.7

*Percentage of cholera cases hospitalised is varied under this scenario.

†The workdays lost are valued at a certain percentage of GDP per capita per day.

GDP, gross domestic productivity.

### Probabilistic multivariate sensitivity analysis

**Figure 1:**
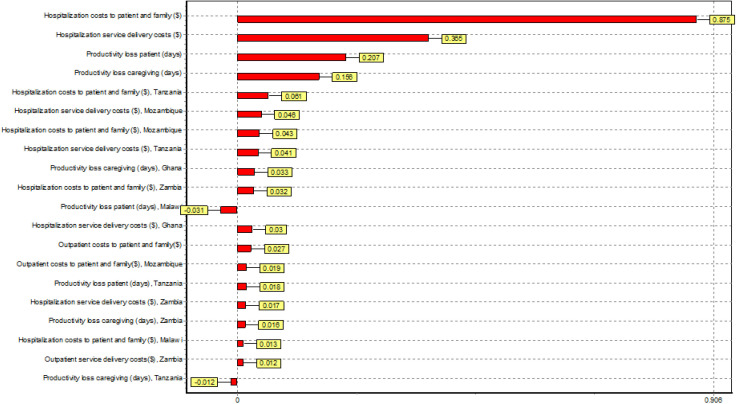
Spearman’s rank correlation coefficient: economic burden of cholera in Africa.

Probabilistic multivariate sensitivity analysis showed OOP costs during hospitalisation, service delivery costs at hospitalisation, and the number of workdays lost among cholera patients and caregivers, have the largest impact on the results (figure 1).

## Discussion

The total economic burden of cholera in Africa was estimated at US$1 billion (I$2.5 billion) which was around US$1.1 per capita for the year 2015, of which only US$0.06 is borne by the public health system. Lost productivity due to premature death due to cholera was estimated to be US$900 (I$2.2 billion) for the year 2015. Productivity loss due to premature death accounted for 87.7% of the total economic burden of cholera in Africa. When costs linked to productivity loss are excluded, 45.6% of the costs were borne by the health system, with the rest borne by affected families including 23.6% as OOP costs and 30.8% as productivity loss due to inability to work. Probabilistic multivariate sensitivity analysis demonstrated that the costs around hospitalisation and loss of workdays are driving the overall costs, which the scenario analysis showed that the economic burden is heavily dependent on how we value loss of workdays.

The Global Task Force for Cholera Control has proposed an ambitious plan to reduce 90% of cholera cases by 2030 as a part of the Ending Cholera—a Global Roadmap to 2030.^25^ This plan needs substantial investment for cholera control including in safe WASH and oral cholera vaccines, in addition to the mapping of cholera hotspots, surveillance, treatment and community engagement. This economic burden estimate may help in expanding thinking beyond a governmental perspective to a wider societal perspective, and whereby many of the African countries may recognise the value of making such investments in cholera control. The economic burden of cholera as estimated here has comparative value for decision-makers on the quantum of investment and its relative return and is also useful for conducting economic evaluation and quantifying the value of such investments.

A previous study on the economic burden of cholera in Africa showed costs of US$39 million to US$156 million, depending on the assumptions on life expectancy (73, 53, 40 years) and year of reported cholera cases (2005, 2006 and 2007).^5^ When converted into 2015 US$ by adjusting for world inflation, the economic burden ranged from US$61.4 million to US$245.5 million. Whereas the current study estimated an economic burden of US$519.3 million to US$1.8 billion based on estimated cholera cases and US$29.4 million based on WHO reported cases in 2015. This was a model-based study and had used standard assumptions. While the current study tries to extrapolate field-based data to estimate the economic burden to around US$1 billion in 2015. Although this study tries to draw inputs from field data, such data have limitations. The biggest driver for the cost difference is the incidence number of cholera cases and deaths. The previous study used cholera incidence deaths as per WHO reports, while the current study uses a comparative scenario analysis (table 2). When WHO reports are used for incidence estimates, the economic burden in the current study is US$29.4 million which is low compared with the previous publication. This is because a larger number of cases and deaths were reported to WHO in 2005–2007 compared with 2015. The number of cases ranged from 125 018 to 2 03 564 and deaths ranged from 2230 to 5281 in 2005–2007 while it was only 71, 16 cases and 736 deaths in 2015.

The economic burden of cholera estimation study in Asia^6^ that had used a comparative methodology based on field data had shown an economic burden of US$987.1 for 2015, which is comparable to the US$1 billion reported here for Africa. However, the number of cholera cases is estimated to be lower in Africa compared with Asia in the base-case (1 008 642 vs 851 396), indicating a relatively higher cost per case in Asia. The average unit cost per case in Asia was US$1159 compared with US$1029 in Africa.

### Limitations

This estimate of the economic burden of cholera used the latest available field-based information in its analysis, but not without limitations.

First, the incidence data on cholera were estimated previously^2^ by extrapolating field-based data; however, these were not real numbers but estimations. While, we also used WHO reported cases and deaths in one scenario which showed a substantially lower economic burden, but even these cannot be considered as real numbers as the WHO reported cases are known to be an underestimate. With cholera incidence numbers being the main cost drivers in this analysis, the potential unknowns in the incidence data and resultant variations in economic burden need to be considered when using the results. A scenario analysis that varied incident cases and deaths are presented to understand this limitation.

Second, the entire cost-of-illness field data inputs came from five African papers, four of which had only costs for hospitalised cases which means that the cost-of-illness data are heavily dependent on either extrapolation or assumptions. It is known that in many African countries cholera treatments are provided through specific centres known as ‘cholera treatment centres’ often set up adjacent to existing health facilities, close to cholera-affected communities. Most severe cases are hospitalised in these centres, while others are kept under observation along with the provision of rehydration and treatment. This situation creates a challenge in differentiating the typical categorisation of cholera cases as hospitalised and outpatient cases, with most studies resulting in reporting everyone as hospitalised. The health facility costs of such specialised treatment centres could be different from typical facility-based service provision. In the absence of outpatient data, we have used WHO-CHOICE data that may not represent typical cholera treatment situations. Because the base-case assumes 90% of cholera cases are hospitalised, this bias is minimised. In addition, a scenario analyses with a range of hospitalisation rates was presented.

Third, OOP costs and the number of workdays lost are collected from patient and family surveys which are subject to many biases. The OOP costs are different in different countries, and range depending on the patient’s location, type of health facility, travel time and distance, and the healthcare system. It is also subject to the limitations of surveys such as how and when the data was collected. This data was extrapolated to all African countries from five study sites. The extrapolation methods may have resulted in underestimation or overestimation of OOP costs, the direction of which is difficult to ascertain. Extensive sensitivity analyses are conducted to understand these limitations.

Fourth, the overall economic burden is heavily dependent on how the lost workdays and years lost due to death are valued. Cholera is known to affect economically marginalised people more often, and a GDP per capita estimation of valuing their lost time could be an overestimation. Also, some of the people may not be in the workforce, may not earn in the future, but still, their time is valued. If an ill worker is replaced, it may affect family income but may not affect the overall economy. Thus, the potential income loss presented in this analysis need not represent the true income loss in African countries. To understand these limitations a scenario analysis where lost workdays and death years are valued at 75%, 50% and 25% of GDP per capita.

Lastly, cholera is known to affect the tourism and export industries which are not accounted for here, resulting in an underestimation of the economic burden. Similarly, the costs of outbreak control are not included here. Also, health facility costs do not include diagnostic costs.

### Conclusion

This study estimates the economic burden of cholera in 44 African countries, which can be used as evidence for making the case for cholera control measures such as investments in improving WASH, a better surveillance system, the use of oral cholera vaccination, and other measures described in the Ending Cholera—a Global Roadmap to 2030.^25^ There is some evidence on the cost-effectiveness of using oral cholera vaccines in the control of cholera.^4^ These data can serve as input for conducting economic evaluation such as a cost-effectiveness analysis of using oral cholera vaccine in the Ending Cholera—a Global Roadmap to 203*0*, with additional information such as vaccine delivery costs^26^ and vaccination campaigns.^27^ Study limitations, which include uncertainty related to cholera incidence rates, cholera hospitalisation rates, valuation of lost time and hospitalisation costs, need to be thoroughly considered before using the data for calculating the economic burden of cholera in Africa.

## Supplementary Material

Reviewer comments

Author's manuscript
