# Supplementary material for: Model-based estimation of the economic burden of cholera in Africa
Source: BMJ Open. 2021 Mar 23;11(3):e044615. doi: 10.1136/bmjopen-2020-044615 (PMC7993295; doi:10.1136/bmjopen-2020-044615)
Supplement: Supplementary data [file bmjopen-2020-044615supp001.pdf]

Annex

***Annex1**: Comparison of methodology and results of Kirigia et al (5) and current paper*

Parameter	Kirigia et al (5)	Current paper
Year of analysis (disease burden)	2006, 2007, 2008	2015
Year of price	2002	2015
Data source for cholera cases	WHO reported	Global burden estimate and WHO reported
Household costs	Average (non-cholera) health care costs reported in WHO survey in 16 countries	Average cholera specific household costs reported in 5 cost of illness studies
Health system costs	The hotel were assumptions based on non-cholera cases reported by WHO. The medicines and diagnostic costs were estimated based on standard practices using assumptions.	Average of reported health system costs from 5 cost of illness studies for hospitalized cases and from WHO database of outpatients. Diagnostic costs are not included.
Indirect costs	Productivity loss for the duration of illness and premature deaths were valued by gross national income per capita	Productivity loss for the duration of illness and premature deaths were valued by gross domestic product per capita
Scenario analysis	For the year (2006, 2007, 2008), Life expectancy (73, 53, 40 years)	Disease burden (3 estimates and WHO reported), Hospitalization rate (90%, 75%, 55%), GDP per capita (100%, 75%, 50%, 25%)
Range of estimated economic burden in $2015 including mortality costs	$61.4 million to $245.5 million ($39 million and $156 million in 2002 prices)	$519.3 million to $1.8 billion based on estimated cases and $29.4 million based on WHO reported cases

(5) Kirigia JM, Sambo LG, Yokouide A, Soumbey-Alley E, Muthuri LK, Kirigia DG. Economic burden of cholera in the WHO African region. BMC international health and human rights. 2009;9:8.

Annex 2: Economic burden of cholera in Africa by country

Country*	Population in 2015 (2)	GDP per capita US$ 2015 (18)	GDP per capita I$ 2015 (18)	Infant mortality rate per 1000 live births (28)	Life expectancy at birth 2015(21)	Current health expenditure per capita (in 2015) (29)	Cases in 2015(2)	Deaths in 2015(2)	Productivity loss due to illness (US$2015)	Public health system cost (US$2015)	Out of pocket costs (US$2015)	Economic burden excluding deaths (US$2015)	Average cost of illness (US$2015)	Productivity loss due to premature deaths (US$2015)	Total economic burden (US$2015)	Cholera economic burden per capita in US$ 2015
Angola	27,884,380	$4,355	$7,412	57.8	61.2	$108.68	19,158	728	$2,225,925	$1,132,539	$792,875	$4,151,339	$216.69	$69,296,429	$73,447,768	$2.63
Benin	10,575,962	$1,077	$2,889	64.4	60.6	$ 31.29	4,755	181	$136,614	$277,839	$196,785	$611,239	$128.55	$4,209,382	$4,820,620	$0.46
Burkina Faso	18,110,616	$576	$1,704	53.6	59.9	$33.51	6,527	248	$100,233	$381,155	$270,122	$751,509	$115.14	$3,049,953	$3,801,463	$0.21
Burundi	10,160,034	$294	$769	45.8	57.1	$14.39	10,341	393	$81,249	$602,779	$427,970	$1,111,998	$107.53	$2,341,441	$3,453,440	$0.34
Cameroon	23,298,376	$1,339	$3,469	55.7	57.6	$63.63	9,487	361	$338,811	$555,189	$392,627	$1,286,627	$135.62	$9,873,360	$11,159,987	$0.48
Cape Verde	524,740	$2,997	$6,313	18.3	72.6	$159.22	117	4	$9,356	$6,900	$4,843	$21,099	$180.30	$338,692	$359,792	$0.69
Central Africa Rep.	4,493,171	$354	$698	90.7	51.4	$21.98	5,742	218	$54,200	$335,138	$237,633	$626,971	$109.19	$1,362,506	$1,989,477	$0.44
Chad	14,110,971	$947	$2,648	76.5	52.6	$35.52	11,486	436	$290,170	$671,161	$475,372	$1,436,704	$125.08	$7,527,860	$8,964,564	$0.64
Comoros	777,435	$1,260	$2,637	56	63.5	$58.24	68	3	$2,296	$3,992	$2,827	$9,115	$133.44	$74,080	$83,195	$0.11
Congo, Democratic Republic	76,244,532	$436	$760	73.9	59.2	$20.11	136,821	5,199	$1,591,458	$7,976,946	$5,662,446	$15,230,850	$111.32	$47,788,731	$63,019,580	$0.83
Congo, Rep.	4,856,093	$2,067	$7,318	38.9	64.1	$58.45	4,770	181	$263,037	$280,720	$197,393	$741,150	$155.39	$8,569,186	$9,310,337	$1.92
Cote d'voire	23,226,148	$1,397	$3,427	64.8	53.1	$75.45	15,181	577	$565,934	$888,232	$628,278	$2,082,444	$137.17	$14,868,449	$16,950,893	$0.73
Djibouti	913,998	$2,533	$4,611	54.2	62.3	$81.73	164	5	$11,091	$9,603	$6,793	$27,487	$167.46	$295,674	$323,160	$0.35
Eritrea	3,342,818	$352	$950	34.1	64.6	$27.18	12,860	489	$120,883	$750,166	$532,229	$1,403,279	$109.12	$3,968,339	$5,371,618	$1.61
Ethiopia	100,835,453	$703	$1,811	43.8	65.0	$25.11	195,093	7,414	$3,657,892	$11,379,858	$8,074,096	$23,111,846	$118.47	$120,778,513	$143,890,360	$1.43
Gabon	1,947,690	$7,453	$17,970	36.3	65.7	$197.94	405	15	$80,456	$24,265	$16,745	$121,467	$300.20	$2,680,703	$2,802,169	$1.44
Gambia, The	2,085,860	$650	$2,375	41.9	61.0	$21.85	370	14	$6,407	$21,581	$15,302	$43,290	$117.08	$198,500	$241,790	$0.12
Ghana	27,849,203	$1,754	$5,524	38.9	62.4	$82.41	6,794	258	$383,782	$439,711	$524,804	$1,348,298	$198.45	$10,087,937	$11,436,235	$0.41
Guinea	11,432,096	$712	$1,838	68.5	59.4	$42.25	5,656	215	$107,438	$329,968	$234,059	$671,465	$118.73	$3,239,028	$3,910,493	$0.34
Guniea-Bissau	1,737,207	$644	$1,679	59.5	57.0	$ 38.14	1,142	43	$19,619	$66,610	$47,263	$133,492	$116.89	$559,084	$692,575	$0.40
Kenya	47,878,339	$1,453	$3,235	33.3	66.7	$66.02	67,091	2,549	$2,601,329	$3,920,223	$2,776,614	$9,298,166	$138.59	$87,849,671	$97,147,837	$2.03

Lesotho	2,059,011	$1,167	$3,174	71.2	53.7	$97.47	1,768	67	$55,025	$103,310	$73,170	$231,505	$130.94	$1,466,453	$1,697,959	$0.82
Liberia	4,472,229	$764	$1,421	58.5	62.0	$71.94	2,137	81	$43,582	$124,613	$88,441	$256,636	$120.09	$1,370,686	$1,627,323	$0.36
Madagascar	24,234,080	$400	$1,470	40.8	65.5	$22.13	22,766	865	$243,165	$1,328,860	$942,189	$2,514,214	$110.44	$8,084,510	$10,598,724	$0.44
Malawi	16,745,305	$354	$1,129	40.3	62.7	$34.00	10,209	388	$118,651	$718,229	$267,074	$1,103,954	$108.14	$3,068,258	$4,172,211	$0.25
Mali	17,438,772	$773	$2,115	67.2	57.5	$30.83	10,070	383	$207,579	$588,249	$416,755	$1,212,582	$120.42	$6,033,505	$7,246,088	$0.42
Mauritania	4,046,304	$1,303	$4,297	55.4	63.1	$53.61	3,609	137	$125,505	$211,067	$149,361	$485,933	$134.64	$4,021,206	$4,507,139	$1.11
Mozambique	27,042,001	$529	$1,195	59.3	57.7	$27.38	50,811	1,931	$655,324	$2,409,795	$1,875,380	$4,940,499	$97.23	$20,923,973	$25,864,472	$0.96
Namibia	2,314,901	$5,160	$11,320	33.5	63.8	$466.11	610	23	$83,980	$36,017	$25,245	$145,241	$238.10	$2,701,262	$2,846,503	$1.23
Niger	20,001,663	$363	$964	52	59.7	$25.72	16,212	616	$156,885	$945,879	$670,946	$1,773,710	$109.41	$4,750,822	$6,524,533	$0.33
Nigeria	181,137,454	$2,726	$6,053	78.7	53.0	$97.52	134,155	5,098	$9,758,067	$7,856,671	$5,552,111	$23,166,849	$172.69	$255,879,611	$279,046,459	$1.54
Rwanda	11,369,066	$736	$1,907	30.8	66.7	$46.40	15,171	577	$298,052	$885,490	$627,864	$1,811,406	$119.40	$10,076,243	$11,887,649	$1.05
São Tomé and Principe	199,439	$1,570	$3,033	27	66.5	$86.91	39	1	$1,633	$2,282	$1,614	$5,529	$141.78	$37,118	$42,647	$0.21
Senegal	14,578,450	$1,187	$3,118	35.1	66.8	$50.96	7,252	276	$229,646	$424,220	$300,130	$953,996	$131.55	$7,777,910	$8,731,906	$0.60
Sierra Leone	7,171,909	$600	$1,433	87.8	51.4	$119.82	5,177	197	$82,813	$302,022	$214,254	$599,089	$115.72	$2,085,448	$2,684,538	$0.37
Somalia	13,797,204	$293	$775	83.1	55.9	NA	11,220	359	$87,847	$656,748	$464,349	$1,208,944	$107.75	$2,076,914	$3,285,858	$0.24
South Sudan	10,715,657	$1,225	$2,002	63.7	56.3	$28.05	17,098	650	$558,887	$1,000,988	$707,614	$2,267,490	$132.62	$15,851,280	$18,118,769	$1.69
Sudan	38,902,948	$1,698	$4,282	45.4	64.3	$158.02	26,311	842	$1,191,987	$1,540,819	$1,088,902	$3,821,708	$145.25	$32,772,322	$36,594,030	$0.94
Swaziland	1,104,038	$3,780	$10,038	47.9	57.1	$231.42	1,384	53	$139,559	$81,285	$57,278	$278,122	$200.96	$4,053,661	$4,331,783	$3.92
Tanzania	51,482,638	$948	$2,782	41.9	65.0	$32.09	84,550	3,213	$1,945,796	$5,254,900	$2,174,112	$9,374,807	$110.88	$70,515,725	$79,890,533	$1.55
Togo	7,323,162	$564	$1,546	51.4	59.9	$35.21	4,919	187	$73,986	$287,293	$203,577	$564,855	$114.83	$2,253,130	$2,817,985	$0.38
Uganda	38,225,447	$647	$2,272	38.6	59.6	$39.77	38,066	1,446	$656,973	$2,222,025	$1,575,392	$4,454,389	$117.02	$19,855,976	$24,310,365	$0.64
Zambia	15,879,370	$1,310	$3,844	44.5	61.4	$58.52	20,618	784	$749,934	$1,203,369	$440,605	$2,393,908	$116.11	$22,512,900	$24,906,808	$1.57
Zimbabwe	13,814,642	$1,425	$2,640	38.5	60.4	$94.29	10,462	398	$397,751	$612,289	$432,978	$1,443,018	$137.93	$12,217,700	$13,660,718	$0.99
Total/average	936,340,81			52.26	60.61		1,008,642	38,104	$30,510,80	$58,850,99	$39,866,41	$129,228,2		$909,344,1	$1,038,572,	$39.20

	2								7	3	8	18		33	351	
