# Supplementary material for: Model-based estimation of the economic burden of cholera in Africa
Source: BMJ Open. 2021 Mar 23;11(3):e044615. doi: 10.1136/bmjopen-2020-044615 (PMC7993295; doi:10.1136/bmjopen-2020-044615)
Supplement: Author's manuscript [file bmjopen-2020-044615.draft_revisions.pdf]

BMJ Open

BMJ Open is committed to open peer review. As part of this commitment we make the peer review history of every article we publish publicly available.

When an article is published we post the peer reviewers' comments and the authors' responses online. We also post the versions of the paper that were used during peer review. These are the versions that the peer review comments apply to.

The versions of the paper that follow are the versions that were submitted during the peer review process. They are not the versions of record or the final published versions. They should not be cited or distributed as the published version of this manuscript.

BMJ Open is an open access journal and the full, final, typeset and author-corrected version of record of the manuscript is available on our site with no access controls, subscription charges or pay-per-view fees (http://bmjopen.bmj.com).

If you have any questions on BMJ Open's open peer review process please email info.bmjopen@bmj.com

An estimation of the economic burden of Cholera in Africa

Journal:	*BMJ Open*
Manuscript ID	bmjopen-2020-044615
Article Type:	Original research
Date Submitted by the Author:	09-Sep-2020
Complete List of Authors:	Mogasale, Vittal; International Vaccine Institute, Policy and Economic Research<br>Ngogoyo, Samuel ; County government of Nyandarua, Department of Health<br>Mogasale, Vijayalaxmi; Yenepoya Medical College Hospital, Department of Pediatrics
Keywords:	Health economics < HEALTH SERVICES ADMINISTRATION & MANAGEMENT, Health policy < HEALTH SERVICES ADMINISTRATION & MANAGEMENT, Public health < INFECTIOUS DISEASES

I, the Submitting Author has the right to grant and does grant on behalf of all authors of the Work (as defined in the below author licence), an exclusive licence and/or a non-exclusive licence for contributions from authors who are: i) UK Crown employees; ii) where BMJ has agreed a CC-BY licence shall apply, and/or iii) in accordance with the terms applicable for US Federal Government officers or employees acting as part of their official duties; on a worldwide, perpetual, irrevocable, royalty-free basis to BMJ Publishing Group Ltd ("BMJ") its licensees and where the relevant Journal is co-owned by BMJ to the co-owners of the Journal, to publish the Work in this journal and any other BMJ products and to exploit all rights, as set out in our licence.

The Submitting Author accepts and understands that any supply made under these terms is made by BMJ to the Submitting Author unless you are acting as an employee on behalf of your employer or a postgraduate student of an affiliated institution which is paying any applicable article publishing charge ("APC") for Open Access articles. Where the Submitting Author wishes to make the Work available on an Open Access basis (and intends to pay the relevant APC), the terms of reuse of such Open Access shall be governed by a Creative Commons licence – details of these licences and which Creative Commons licence will apply to this Work are set out in our licence referred to above.

Other than as permitted in any relevant BMJ Author's Self Archiving Policies, I confirm this Work has not been accepted for publication elsewhere, is not being considered for publication elsewhere and does not duplicate material already published. I confirm all authors consent to publication of this Work and authorise the granting of this licence.

For peer review only

A model-based estimation of the economic burden of Cholera in Africa

*Vittal Mogasale [a] *, Samuel Mwaura Ngogoyo [b], Vijayalaxmi V Mogasale [c]*

[a] Policy and Economic Research Department, International Vaccine Institute, Seoul, South Korea.

[b] County Government of Nyandarua. Department of Health, Ol Kalou, Kenya.

[c] Department of Pediatrics, Yenepoya Medical College and Research Center, Mangalore, India

***Corresponding author:**

Dr. Vittal Mogasale, Policy and Economic Research Department, International Vaccine Institute, SNU Research Park, 1 Gwanak-ro, Gwanak-gu Seoul 151-742. South Korea. Tel: +82 2 881 1379(Dir); +82 2872 2801 (Ext 379); Fax: + 82 2 872 2803

E-mail: vmogasale@ivi.int; vmogasale@gmail.com

For peer review only

Abstract

Objectives

To estimate the economic burden of cholera in Africa using the latest available data.

Settings

Cholera affected 44 countries in Africa.

Participants

The analysis used data from various public sources including data from cholera cost-of-illness studies conducted in African countries published until September 2019.

Methods

Based on existing data collected from field-based cost-of-illness studies, estimated cholera incidence rates, and reported cholera cases to WHO, this research estimates the economic burden of cholera in Africa from a societal perspective with 2015 as the base year. The cholera economic burden estimate included out-of-pocket costs, public health system costs, productivity loss related to illness, and an optional productivity loss related to premature deaths valued by the human capital approach. As various input data such as cholera incidence, hospitalization rates, and the number of workdays lost were not well defined, a series of scenario analyses and uncertainty analyses, accounting for unknowns and data variability, was conducted. Similarly, the value of time lost due to illness and deaths using the human capital approach was explored through scenario analyses.

Results

In 2015, an estimated 1,008,642 cases in 44 African countries resulted in an economic burden of US$130 million from cholera-related illness and its treatment. When the estimated 38,104 cholera deaths were included in the analysis, the economic burden increased to US$1 billion or international $2.4 billion for the same year. At the same time, when only the 71,126 cases and 937 deaths reported to the WHO are considered, the economic burden was only US$68 million for the year 2015. The estimates of economic burden are thus heavily dependent on the cholera incidence rate, how time lost due to illness and deaths are calculated, hospitalization rates, and hospitalization costs.

Conclusion

For peer review only - http://bmjopen.bmj.com/site/about/guidelines.xhtml

The findings from this study can be used as an economic justification for cholera control in Africa and for generating value-for-money evidence to underpin *Ending Cholera – A Global Roadmap to 2030*. However, study limitations such as the uncertainty in cholera incidence and hospitalization rates should be carefully considered before making decisions.

Strengths and limitations of this study

- Model-based estimation of the economic burden of cholera in 44 African countries was conducted using various secondary data sources.
- The average inpatient health system costs, out of pocket expenses, and the number of days of productivity lost related to cholera treatment from five African papers that presented data was directly applied to countries that did not have data.
- The estimated number of cholera cases was derived from the global burden of disease study and the World Health Organization report.
- The productivity loss related to cholera illness and premature deaths were valued by the human capital approach using gross domestic product per capita.
- Cholera incidence rates, cholera hospitalization rates, and cholera hospitalization costs are the most uncertain parameters that drove the results.

Key Words

cholera, economic burden, Africa, costs, cholera elimination

For peer review only - http://bmjopen.bmj.com/site/about/guidelines.xhtml

Introduction

Cholera, an acute, diarrheal illness caused by infection of the intestine with the toxigenic bacterium *Vibrio cholerae* is linked to poor water, sanitation, and hygiene (WASH) conditions (1). In cholera-endemic countries, an estimated 2.8 million cases and 91,000 deaths occur annually, due to cholera (2). However, the actual number of reported cases to the World Health Organization (WHO) is significantly lower. Globally, around 132,000 cases and 2400 deaths were reported to the WHO in 2016 (3). Cumulatively, from 2000 to 2016, 3.4 million cholera cases and 65,000 deaths worldwide were reported to WHO (3). Countries in Africa continue to experience a disproportionately high number of cholera outbreaks resulting in a high burden of disease and deaths. In 2016, seventeen countries in Africa accounted for 71,000 cases (54% of global cases) with 1,760 deaths (42%of deaths globally) (3). Furthermore, the average case fatality rate of 2.5% in African countries is substantially higher than the global average of 1.8% (3).

When measured as an economic burden, cholera has a significant economic impact on health systems and individuals. The societal cost per cholera case is believed to be more than $1,000 when the wider socio-economic costs such as cost to the health system, the patient's family, and income loss due to death, are taken into account (4). While several studies have been done in various countries on the economic burden of cholera, the cost-effectiveness of interventions, and other related areas of interest, there is a lack of any recent studies estimating the economic burden in Africa looking into the areas of loss of productivity, hospitalization and outpatient costs, quantified at the national and regional levels. A model-based estimate in 2007 reported an annual economic loss ranging from US$39 million to US$64.2 million as a result of cholera in $2002 prices and based on regional life expectancies ranging from 40 to 73 years in Africa (5). The study used several cost assumptions around standard treatment and diagnostic practices which may not be the current situation in Africa and may be outdated.

A recent economic burden estimate in Asia used the actual estimated cost of illness collected from countries to project the economic burden while using a variety of scenario and sensitivity analyses to demonstrate the uncertainties around the estimated and reported cholera cases to WHO (6). This kind of economic burden estimate provides a better understanding of economic offsets that can be achieved through cholera control measures and can be used as evidence to make a case for the effectiveness of cholera control. We used the same methodological approach as in Asia (6) and used the latest data available from field settings in Africa to quantify the economic burden of cholera in Africa.

For peer review only - http://bmjopen.bmj.com/site/about/guidelines.xhtml

Methods

The research involved the selection of countries, identification of cost-of-illness studies to define out-of-pocket expenses, productivity loss, and health system costs to estimate and extrapolate the economic burden, and finally, conduct sensitivity analysis and scenario analyses. Patients or the public were not involved in the design, or conduct, or reporting, or dissemination plans of this research.

Conceptual framework

The primary input needed for estimating the economic burden is the cost-of-illness studies conducted around cholera cases at health facilities in African countries. Such studies provide three sets of key information (1). Out-of-pocket (OOP) expenditures borne by patients and families for cholera treatment which could include medical costs such as medicines and intravenous fluids, or non-medical costs expenditures on the transport and food (2). Loss of income, which is often referred to as productivity costs related to illness, because the patient is unable to work and someone needs to take care of the patient. This is often presented in the number of workdays lost (3). Cost to the health system for providing the cholera treatment which includes costs related to staff, provision of services, maintenance of hospital facilities, etc.

The cost-of-illness data is typically not available from most African countries. In such situations, cost-of-illness was extrapolated to African countries using available data from field studies in the region (out of pocket expenses, inpatients costs, and productivity loss) and WHO estimates (outpatient costs) such that each country has a unit cost of illness (cost of illness per cholera case). When the total number of cholera cases is multiplied by the unit cost-of-illness, the economic burden for that country is derived which is summated at the regional level. However, all the input costs and the number of cases are not constant numbers. There may be several biases and reported variations such as under-reporting or over-estimations because of which we conduct a series of scenario analyses and uncertainty analyses as described below so that the limitations and boundaries are well understood.

Country selection and disease burden estimation

African countries as defined by the United Nations (7), that either reported cholera cases to WHO or were estimated to have cholera cases, were selected for the analysis. The year 2015 was selected as the reference year for data comparability purposes since both, disease burden estimates (2) and cases reported to WHO (8), were available for that year. Most often than not, cholera is under-reported by many countries because of reasons such as the lack of diagnostics and reagents to test cholera, inadequacies in the surveillance system, differences in case definition, and the reluctance of authorities to acknowledge or report cholera (9). Therefore, the data in the WHO report is under-reported and an economic burden

For peer review only - http://bmjopen.bmj.com/site/about/guidelines.xhtml

estimate based on the WHO report will be an underestimation. Whereas the 2015 global burden of disease estimate (2) has considered several incidence scenarios as part of sensitivity analysis, of which three were used in this analysis. In total, four cholera incidence scenarios were used in the analysis as listed below.

(a) Base case: The Global Burden of Cholera study (2) had extrapolated the cholera incidence data from Beira, Mozambique (10) to the rest of Africa after applying the incidence rate to the population at risk based on the percentage of people without sustainable access to improved water. We used the same incidence numbers in the base case. This methodology assumes that only a part of the population is at risk for cholera. This was one of the sensitivity analyses in the Global Burden of Cholera study (2).

(b) Liberal case: The incidence rate from Beira, Mozambique was extrapolated to the rest of Africa after applying an incidence correction assumption by African WHO mortality strata D (AFR-D) (11). It was assumed that AFR-D had half the incidence as Beira, while the rest of African countries were assumed to have the same incidence rate as Beira (2). Here the assumption is that the whole of the population is at risk for cholera, but with different incidence rates. This was the base case in the Global Burden of Cholera publication (2).

(c) Conservative case: As other sensitivity analyses used in the 2015 global burden of disease estimate produced a similar number of cholera cases in Africa, a conservative estimate was derived by applying half of the incidence rate as the base case.

(d) WHO report case: A separate scenario analysis was conducted based on the number of cholera cases and deaths reported to WHO in 2015.

The 2015 global burden of disease study (2) had applied a 3.8% case fatality rate to estimate death, and the same was applied in the first three scenario analyses.

Cholera cost-of-illness

As described previously in the conceptual framework, there are three types of costs: out-of-pocket costs, productivity loss, and health system costs. These costs were extracted from literature based on actual studies conducted in healthcare settings. A previous systematic literature review (4) had identified two cost-of-illness studies conducted in Africa, one in Mozambique (12) and another in Tanzania (13). Furthermore, a PubMed search in September 2019 yielded three more studies published subsequently --

For peer review only

one each from Malawi (14), Ghana (15), and Zambia (16). Data from these 5 studies were extracted and used as inputs in the analysis.

Out-of-Pocket (OOP) costs

The OOP costs for hospitalized cases were reported for all five papers used in the study, while only Malawi reported the OOP costs for outpatient cases. There are two types of OOP costs, direct medical, and direct non-medical costs. Direct medical costs include expenditure accrued by patients for diagnosis, medicines, and other costs directly related to the treatment of cholera. Direct non-medical costs include expenditures on travel to the healthcare facility, room and boarding, food, and other costs not directly related to treatment. All the costs reported in the different years were converted to 2015 US dollars by adjusting them based on annual percent changes in world consumer prices as reported by the International Monitory Fund (17). For countries that did not report OOP costs either for hospitalization or outpatient, the average cost derived from the rest of the reported costs were applied directly.

Lost productivity

Loss of productivity due to illness was estimated based on the average number of lost workdays for patients and caregivers throughout the illness and the recuperation period documented separately for patients and caregivers. Lost workdays for Ghana, Malawi, and Mozambique were extracted directly from their respective sources (12, 14, 15). Data from Tanzania only includes the duration of illness and does not account for the loss of productivity during the recuperation period (13). While the study in Zambia did not give an exact average, we were able to estimate the average based on the survey results (16). The ranges of the lost days were calculated based on the range derived from the study in Tanzania, where the variability of 20% was observed. The lost days were then multiplied by the average gross domestic productivity (GDP) per capita (18) to derive total productivity loss per cholera episode. As cholera incidence may be higher in people with low income, we used three scenarios that estimated productivity loss at 75%, 50%, and 25% of GDP per capita.

Health system costs

Service delivery costs were also extracted from the papers for each hospitalized and outpatient cases. Health facility costs for service delivery included personnel, medicines, diagnostic tools, medical equipment, infrastructure, beds, and utilities that patients are not responsible for paying. Of the five publications used in this study, Malawi, Tanzania, and Mozambique reported health service delivery costs for hospitalized cases. For countries without reported costs, the mean hospitalization health facility costs for these three African countries were applied. Only Malawi reported outpatient service delivery costs.

For peer review only - http://bmjopen.bmj.com/site/about/guidelines.xhtml

For peer review only

The country-specific average outpatient costs for health centers with and without beds from the WHO-CHOICE (19) project were applied to all other countries.

Loss of productivity because of death

To estimate the productivity loss due to premature death from cholera, the mean age of cholera death was derived from a multi-country cholera surveillance project that reported 873 deaths in six African surveillance zones (20). Life expectancy at birth for each country was subsequently subtracted by the mean age of cholera. Information for life expectancy used the 2015 data provided by the World Bank (21). To convert the years lost to monetary values, GDP per capita (18) was multiplied by the number of deaths from cholera, and the number of productive years lost per death. A discount rate of 3% was applied to future earning potential. The GDP per capita although may be considered an overestimate considering cholera may disproportionately affect poor people, it allows accounting for everyone affected equally and their variable life expectancy.

Hospitalization rate

Only the study conducted in Malawi reported a hospitalization rate, at 90% hospitalized and 10% receiving care for less than 12 hours, which is analogous to outpatient care. Other studies included in the analysis were unclear whether all cases were hospitalized, or only hospitalized cases were accounted for. Since 90 to 100% hospitalization is considered high, the analysis was conducted based on three scenarios: (a) under the assumption of 90% hospitalization, (b) average of 75% hospitalization, and (c) average of 55% hospitalization. Assumption (b) was determined based on a previous investment case study (22, 23) while assumption (c) was based on results from an economic burden study conducted for Asian countries (6) which is the average hospitalization rate in Asia.

Probabilistic multi-variate sensitivity analysis

A probabilistic multi-variate sensitivity analysis was conducted using Ersatz (24). A beta-PERT distribution was used for cost inputs which use the minimum, mean, and maximum as input parameters. The beta-PERT distribution is most suited for cost modeling or parameters obtained through the survey that present minimum, a maximum, and most likely values (24). To estimate 95% confidence intervals a Monte Carlo simulation was conducted based on 5,000 random draws for each input parameter based on the distribution allocated.

Scenario Analysis

For peer review only - http://bmjopen.bmj.com/site/about/guidelines.xhtml

Among all input parameters, disease burden (the number of cases) and hospitalization rates had limited information that could be captured in one single number with uncertainty ranges. Therefore, scenario analyses were conducted for both disease burden and hospitalization rates. Disease burden scenarios included three estimates as explained earlier and is based on a previous publication (2) and the reported cholera cases and deaths to WHO (8). Hospitalization rate had three scenarios as described above namely 90%, 75%, and 55% hospitalization rates. As productivity loss is driving overall costs, we conducted scenario analysis by valuing loss of workdays at 75%, 50%, and 25% of GDP per capita.

All the costs were converted to their equivalent value in 2015 US dollars (US$). For the conversion, the annual percent changes in world consumer prices as established by the IMF were applied (17). The results were presented both in 2019 US$ and 2019 International dollars (I$). For the conversion from US$ to I$, a country-specific multiplication factor from World Bank data that ranged from 1.63 to 3.67 was used (18).

The comparison of methodology with previous economic burden study

The previous cholera economic burden study estimated the costs in 2002 prices for WHO reported cholera cases in 2005, 2006, and 2007 (5). It used WHO data sources and assumptions based on standard clinical practices for estimating health system (hotel cost, diagnostic costs, and medicine costs) and out of pocket (household) costs. A methodological comparison table is provided in **Annex 1**. The current study estimated cholera economic burden in Africa using available data from the field-based cost of illness study and most recent disease burden estimates available and in a comparable approach to economic burden estimate in Asia (6).

Patient and public involvement

Patients and/or the public were not involved in this research.

Results

Based on a literature review, average workday loss of 5.76 (range 2.96 to 9.60) and 3.86 (range 3.09 to 4.64) was estimated for cholera cases and caregivers respectively, irrespective of their hospitalization status **(Table 1)**. The OOP costs were US$ 38.87 (range $6.07 to $109.94) for hospitalized cases and US$ 5.71 (range $0.89 to $16.15) for those who visited the health facility but did not get hospitalized. The health facility costs were estimated at US$ 65.77 and US$ 2.84 for hospitalized and outpatient cases respectively. The mean age of death due to cholera was 23.74 years. The analysis included 44 African countries and the list of countries included in the analysis is available in **Annex 2**.

For peer review only - http://bmjopen.bmj.com/site/about/guidelines.xhtml

In the base case analysis, 1,008,642 cases of all ages were included from 44 African countries, while 71,176 cholera cases from 16 countries were reported to WHO (**Annex 2**). The number of deaths estimated in the base case was 38,104 from 44 countries, while 937 were reported to WHO. Excluding deaths, the total economic burden is US$130 million and I$ 315 million for the year 2015 (**Table 2**). When productivity losses related to deaths are taken into account, the total economic burden increases to US$1 billion, and I$2.4 billion for the same year. Country-specific economic burden and unit costs are provided in **Annex 2** which shows the per capita economic burden of cholera ranges from US$0.1 to US$3.9 with the average at US$1.1.

Scenario analyses

The scenario analyses showed that when assumptions on the number of cholera cases changed, the economic burden excluding death ranged from $64.6 million to $227.2 million. The estimated/ reported number of cases influences the economic burden considerably and drops to $8.6 million when WHO reported cholera cases are considered in the analysis (**Table 3**).

The scenario analysis that varied hospitalization rates had a relatively small impact on the overall economic burden. When workdays lost are valued at 75%, 50%, and 25% of GDP per capita, a large reduction of economic burden was observed (**Table 4**).

Probabilistic multi-variate sensitivity analysis

Probabilistic multi-variate sensitivity analysis showed OOP costs during hospitalization, service delivery costs at hospitalization, and the number of workdays lost among cholera patients and caregivers, have the largest impact on the results (**Figure 1**).

Discussion

The total economic burden of cholera in Africa was estimated at US$1 billion (I$2.5 billion) which was around US$1.1 per capita for the year 2015, of which only US$0.06 is borne by the public health system. Lost productivity due to premature death due to cholera was estimated to be US$900 (I$2.2 billion) for the year 2015. Productivity loss due to premature death accounted for 87.7% of the total economic burden of cholera in Africa. When costs linked to productivity loss are excluded, 45.6% of the costs were borne

For peer review only - http://bmjopen.bmj.com/site/about/guidelines.xhtml

by the health system, with the rest borne by affected families including 23.6% as OOP costs and 30.8% as productivity loss due to inability to work. Probabilistic multi-variate sensitivity analysis demonstrated that the costs around hospitalization and loss of workdays are driving the overall costs, which the scenario analysis showed that the economic burden is heavily dependent on how we value loss of workdays.

The Global Task Force for Cholera Control (GTFCC) has proposed an ambitious plan to reduce 90% of cholera cases by 2030 as a part of the *Ending Cholera- a Global Roadmap to 2030* (25). This plan needs substantial investment for cholera control including in safe water, sanitation and hygiene, and oral cholera vaccines, in addition to the mapping of cholera hotspots, surveillance, treatment, and community engagement. This economic burden estimate may help in expanding thinking beyond a governmental perspective to a wider societal perspective, and whereby many of the African countries may recognize the value of making such investments on cholera control. The economic burden of cholera as estimated here has comparative value for decision-makers on the quantum of investment and its relative return and is also useful for conducting economic evaluation and quantifying the value of such investments.

A previous study on the economic burden of cholera in Africa showed costs of US$39 million to US$156 million, depending on the assumptions on life expectancy (73, 53, 40 years) and year of reported cholera cases (2005, 2006 and 2007) (5). When converted into 2015 US$ by adjusting for world inflation, the economic burden ranged from $61.4 million to $245.5 million. Whereas the current study estimated an economic burden of $519.3 million to $1.8 billion based on estimated cholera cases and $29.4 million based on WHO reported cases in 2015. This was a model-based study and had used standard assumptions. While the current study tries to extrapolate field-based data to estimate the economic burden to around 1 billion USD in 2015. Although this study tries to draw inputs from field data, such data has limitations. The biggest driver for the cost difference is the incidence number of cholera cases and deaths. The previous study used cholera incidence deaths as per WHO reports, while the current study uses a comparative scenario analysis (Table 2). When WHO reports are used for incidence estimates, the economic burden in the current study is US$29.4 million which is low compared to the previous publication. This is because a larger number of cases and deaths were reported to WHO in 2005-2007 compared to 2015. The number of cases ranged from 125,018 to 203,564 and deaths ranged from 2,230 to 5,281 in 2005-2007 while it was only 71,16 cases and 736 deaths in 2015.

The economic burden of cholera estimation study in Asia (6) that had used a comparative methodology based on field data had shown an economic burden of US $987.1 for 2015, which is comparable to the US$ 1 billion reported here for Africa. However, the number of cholera cases is estimated to be lower in

For peer review only - http://bmjopen.bmj.com/site/about/guidelines.xhtml

Africa compared to Asia in the base-case (1,008,642 vs 851,396), indicating a relatively higher cost per case in Asia. The average unit cost per case in Asia was US$1,159 compared to US$1,029 in Africa.

Limitations

This estimate of the economic burden of cholera used the latest available field-based information in its analysis, but not without limitations.

Firstly, the incidence data on cholera were estimated previously (2) by extrapolating field-based data; however, these were not real numbers but estimations. While, we also used WHO reported cases and deaths in one scenario which showed a substantially lower economic burden, but even these cannot be considered as real numbers as the WHO reported cases are known to be an underestimate. With cholera incidence numbers being the main cost drivers in this analysis, the potential unknowns in the incidence data and resultant variations in economic burden need to be considered when using the results. A scenario analysis that varied incidence cases and deaths are presented to understand this limitation.

Secondly, the entire cost-of-illness field data inputs came from five African papers, four of which had only costs for hospitalized cases which means that the cost-of-illness data is heavily dependent on either extrapolation or assumptions. It is known that in many African countries cholera treatments are provided through specific centers known as 'cholera treatment centers' often set up adjacent to existing health facilities, close to cholera-affected communities. Most severe cases are hospitalized in these centers, while others are kept under observation along with the provision of rehydration and treatment. This situation creates a challenge in differentiating the typical categorization of cholera cases as hospitalized and outpatient cases, with most studies resulting in reporting everyone as hospitalized. The health facility costs of such specialized treatment centers could be different from typical facility-based service provision. In the absence of outpatient data, we have used WHO-CHOICE data that may not represent typical cholera treatment situations. Because the base-case assumes 90% of cholera cases are hospitalized, this bias is minimized. In addition, a scenario analyses with a range of hospitalization rates was presented.

Thirdly, OOP costs and number of workdays lost are collected from patient and family surveys which are subject to many biases. The OOP costs are different in different countries, and range depending upon the patient's location, type of health facility, travel time and distance, and the health care system. It is also subject to the limitations of surveys such as how and when the data was collected. This data was extrapolated to all African countries from 5 study sites. The extrapolation methods may have resulted in

For peer review only

underestimation or overestimation of OOP costs, the direction of which is difficult to ascertain. Extensive sensitivity analyses are conducted to understand these limitations.

Fourthly, the overall economic burden is heavily dependent on how the lost workdays and years lost due to death are valued. Cholera is known to affect economically marginalized people more often, and a GDP per capita estimation of valuing their lost time could be an overestimation. Also, some of the people may not be in the workforce, may not earn in the future, but still, their time is valued. If an ill worker is replaced, it may affect family income but may not affect the overall economy. Thus, the potential income loss presented in this analysis need not represent the true income loss in African countries. To understand these limitations a scenario analysis where lost workdays and death years are valued at 75%, 50%, and 25% of GDP per capita.

Lastly, cholera is known to affect the tourism and export industries which are not accounted for here, resulting in an underestimation of the economic burden. Similarly, the costs of outbreak control are not included here. Also, health facility costs do not include diagnostic costs.

Conclusion

This study estimates the economic burden of cholera in 44 African countries, which can be used as evidence for making the case for cholera control measures such as investments in improving water, sanitation, and hygiene, a better surveillance system, the use of oral cholera vaccination, and other measures described in the *Ending Cholera- a Global Roadmap to 2030* (25). There is some evidence on the cost-effectiveness of using oral cholera vaccines in the control of cholera (4). This data can serve as input for conducting economic evaluation such as a cost-effectiveness analysis of using oral cholera vaccine in the *Ending Cholera- a Global Roadmap to 2030,* with additional information such as vaccine delivery costs (26) and vaccination campaigns (27). Study limitations, which include uncertainty related to cholera incidence rates, cholera hospitalization rates, valuation of lost time, and hospitalization costs, need to be thoroughly considered before using the data for calculating the economic burden of cholera in Africa.

Contribution

VM developed the concept, conducted the final analysis, and wrote the manuscript. SMN assisted in data collection and conducted the initial analysis. VVM assisted in data analysis and writing the manuscript. All authors have reviewed and accepted the final manuscript.

For peer review only - http://bmjopen.bmj.com/site/about/guidelines.xhtml

Funding

This research was conducted by International Vaccine Institute and supported by the Swedish International Development Cooperation Agency [5410054]; the Korea International Cooperation Agency (KOICA)[KOICA-IVI 6/16/2017]; the UK Foreign, Commonwealth, and Development Office, and Wellcome [214662/Z/18/Z]; and Bill & Melinda Gates Foundation [OPP38590]. The International Vaccine Institute acknowledges its donors including the Republic of Korea and the Republic of India.

Acknowledgment

The authors thank Ms. Monica Moon for initial support in data collection and analysis, Mr. Satyajit Sakar for editing support.

Patient and public involvement

Patients and/or the public were not involved in the design, or conduct, or reporting, or dissemination of this research.

Patient consent

Not required

Data sharing statement

All data are presented in the manuscript, no additional data available

References

1. WHO. Cholera. 2020 [cited 2020 April 12]. Available from: https://www.who.int/news-room/fact-sheets/detail/cholera.
2. Ali M, Nelson AR, Lopez AL, Sack DA. Updated global burden of cholera in endemic countries. PLoS Negl Trop Dis. 2015;9(6):e0003832.
3. Cholera, 2016. Releve epidemiologique hebdomadaire / Section d'hygiene du Secretariat de la Societe des Nations = Weekly epidemiological record / Health Section of the Secretariat of the League of Nations. 2017;92(36):521-30.
4. Hsiao A, Hall AH, Mogasale V, Quentin W. The health economics of cholera: A systematic review. Vaccine. 2018;36(30):4404-24.
5. Kirigia JM, Sambo LG, Yokouide A, Soumbey-Alley E, Muthuri LK, Kirigia DG. Economic burden of cholera in the WHO African region. BMC international health and human rights. 2009;9:8.
6. Mogasale V, Mogasale VV, Hsiao A. Economic burden of cholera in Asia. Vaccine. 2020;38 Suppl 1:A160-A6.
7. UN. Composition of macro geographical (continental) regions, geographical sub-regions, and selected economic and other groupings; Geographical region and composition2011 February 02,

For peer review only - http://bmjopen.bmj.com/site/about/guidelines.xhtml

2014 [cited 2014 February 02]. Available from: http://unstats.un.org/unsd/methods/m49/m49regin.htm.
8. Cholera, 2015. Releve epidemiologique hebdomadaire / Section d'hygiene du Secretariat de la Societe des Nations = Weekly epidemiological record / Health Section of the Secretariat of the League of Nations. 2016;91(38):433-40.
9. Ganesan D, Gupta SS, Legros D. Cholera surveillance and estimation of burden of cholera. Vaccine. 2020;38 Suppl 1:A13-A7.
10. Lucas ME, Deen JL, von Seidlein L, Wang XY, Ampuero J, Puri M, et al. Effectiveness of mass oral cholera vaccination in Beira, Mozambique. N Engl J Med. 2005;352(8):757-67.
11. Ali M, Lopez AL, You YA, Kim YE, Sah B, Maskery B, et al. The global burden of cholera. Bull World Health Organ. 2012;90(3):209-18A.
12. Poulos C, Riewpaiboon A, Stewart JF, Clemens J, Guh S, Agtini M, et al. Costs of illness due to endemic cholera. Epidemiology and infection. 2012;140(3):500-9.
13. Schaetti C, Weiss MG, Ali SM, Chaignat CL, Khatib AM, Reyburn R, et al. Costs of illness due to cholera, costs of immunization and cost-effectiveness of an oral cholera mass vaccination campaign in Zanzibar. PLoS Negl Trop Dis. 2012;6(10):e1844.
14. Ilboudo PG, Huang XX, Ngwira B, Mwanyungwe A, Mogasale V, Mengel MA, et al. Cost-of-illness of cholera to households and health facilities in rural Malawi. PloS one. 2017;12(9):e0185041.
15. Awalime DK, Davies-Teye BBK, Vanotoo LA, Owoo NS, Nketiah-Amponsah E. Economic evaluation of 2014 cholera outbreak in Ghana: a household cost analysis. Health Econ Rev. 2017;7(1):45.
16. Tembo T, Simuyandi M, Chiyenu K, Sharma A, Chilyabanyama ON, Mbwili-Muleya C, et al. Evaluating the costs of cholera illness and cost-effectiveness of a single dose oral vaccination campaign in Lusaka, Zambia. PloS one. 2019;14(5):e0215972.
17. IMF. Inflation rate, average consumer prices 2020 [cited 2020 26 April]. Available from: https://www.imf.org/external/datamapper/PCPIPCH@WEO/OEMDC/ADVEC/WEOWORLD.
18. WorldBank. The World Bank data on GDP 2017 [Available from: http://data.worldbank.org/indicator/NY.GDP.MKTP.CD.
19. WHO-CHOICE. Country-specific unit costs. Geneva: CHOosing Interventions that are Cost Effective (WHO-CHOICE), World Health Organization; 2008 [cited 2012 5/01/2012]. Available from: http://www.who.int/choice/country/country_specific/en/index.html.
20. Sauvageot D, Njanpop-Lafourcade BM, Akilimali L, Anne JC, Bidjada P, Bompangue D, et al. Cholera Incidence and Mortality in Sub-Saharan African Sites during Multi-country Surveillance. PLoS Negl Trop Dis. 2016;10(5):e0004679.
21. WorldBank. Life expectancy at birth, total (years) 2017 [cited 2017 25 April]. Available from: http://data.worldbank.org/indicator/SP.DYN.LE00.IN.
22. Ann Levin DD, YE Kim, J Clemens, AL Lopez, M Ali, C Burgess, S Shin, T Wierzba. An Investment Case for the Accelerated Introduction of Oral Cholera Vaccines. Seoul, South Korea; 2012.
23. Mogasale V, Levin A, Maskery B, DeRoeck D, Kim YE, Clemens J, et al. Oral cholera vaccines to control endemic disease: an economic and epidemiological modelling analysis. Lancet. 2013;382(6).
24. Barendregt JJ. Ersatz. 1.1 ed. Brisbane: Epigear International (www.epigear.com); 2010.
25. WHO. The Global Task Force on Cholera Control 2019 [cited 2019 6, October]. Available from: https://www.who.int/cholera/task_force/en/.
26. Mogasale V, Ramani E, Wee H, Kim J. Oral Cholera Vaccination Delivery Cost in Low- and Middle-Income Countries: An Analysis Based on Systematic Review. PLoS Negl Trop Dis. 2016;10(12).
27. Hsiao A, Desai SN, Mogasale V, Exclerc J-L, Digilio L. Lessons learnt from 12 oral cholera vaccine campaigns in resource-poor settings. WHO Bull 2017;95:303–12.

For peer review only - http://bmjopen.bmj.com/site/about/guidelines.xhtml

Tables

Table 1: Input parameter assumptions used in uncertainty analysis

Input parameter	Mean value	Minimum value	Maximum value	Source
Out-of-pocket costs to patient and family for hospitalization	$38.87	$6.05	$109.94	(12-16)
Out-of-pocket costs to patient and family for outpatient cases	$5.71	$0.89	$16.15	(14)
Number of days with the loss of income — cholera cases	5.76 days	4.61 days	6.97 days	(12-16)
Number of days with the loss of income — caregivers	3.86 days	3.09 days	4.64 days	(12, 14, 15)
Public health service delivery costs for hospitalized cases	$65.77	$39.83	$85.41	(12-14)
Public health service delivery costs for outpatient cases	$2.84	$2.55	$3.31	(14)
Age of death due to cholera (years)	23.74	1.00	66.00	(20)
Proportion of cases hospitalized				
Base case	0.90	NA	NA	(12, 14, 15)
Scenario 1	0.75	NA	NA	(22, 23)
Scenario 2	0.55	NA	NA	(6)

Table 2: Economic burden of cholera in Africa

Economic burden	US$ 2015 (in millions)			I$ 2015 (in millions)		
	Mean	95%LCI	95%UCI	Mean	95%LCI	95%UCI
Lost productivity due to illness	$30.7	$22.4	$ 39.1	$72.8	$53.7	$ 92.1
Public health system costs	$58.9	$ 46.4	$70.8	$145.0	$114.8	$173.4
Out-of-pocket costs	$40.1	$16.2	$69.6	$97.6	$40.0	$168.4
Total economic burden excluding deaths	$129.6	$100.3	$164.2	$315.3	$245.0	$397.8
Lost productivity due to premature deaths	$876.2	$293.6	$1,174.8	$2,091.1	$721.7	$2,793.0
Total economic burden including deaths	$1,005.8	$426.4	$1,304.1	$2,406.4	$1,043.9	$3,107.5

For peer review only - http://bmjopen.bmj.com/site/about/guidelines.xhtml

For peer review only

Table 3: Economic burden of cholera in Africa under various cholera incidence scenarios

	US$ 2015				I$ 2015			
	Base estimate	Sensitivity 1	Sensitivity 2	WHO Report	Base estimate	Sensitivity 1	Sensitivity 2	WHO Report
Cases	1,008,642	1,756,703	504,321	71,176	1,008,642	1,756,703	504,321	71,176
Deaths	38,104	66,416	19,052	937	38,104	66,416	19,052	937
Productivity loss due to illness (millions)	$30.7	$53.9	$15.2	$1.7	$72.8	$131.8	$36.1	$4.0
Public health system costs(millions)	$58.9	$103.0	$29.4	$4.1	$145.0	$260.0	$72.5	$9.5
Out of pocket costs (millions)	$40.1	$70.3	$20.0	$2.8	$97.6	$176.2	$49.6	$6.2
Total economic burden excluding deaths (millions)	$129.6	$227.2	$64.6	$8.6	$315.3	$568.0	$157.2	$19.7
Productivity loss due to premature deaths (millions)	$876.2	$1,600.1	$454.7	$20.8	$2091.1	$3,925.1	$1,084.6	$48.1
Total economic burden including deaths (millions)	$1,005.8	$1,827.3	$519.3	$29.4	$2406.4	$4,493.1	$1,241.8	$67.8

WHO= World Health Organization

Table 4: Economic burden of cholera in Africa under various hospitalization and GDP per capita scenarios

	Bases case	Hospitalization rate 1*	Hospitalization rate 2*	GDP 1#	GDP 2#	GDP 3#
Cases	1,008,642	1,008,642	1,008,642	1,008,642	1,008,642	1,008,642
Deaths	38,104	38,104	38,104	38,104	38,104	38,104
Hospitalization rate (%)	90%	75%	55%	90%	90%	90%
Value of workdays as percentage of GDP per capita per day	100%	100%	100%	75%	50%	25%
Productivity loss due to illness (millions USD)	$30.5	$30.5	$30.5	$22.9	$15.3	$7.6
Public health system costs (millions USD)	$58.9	$49.9	$38.0	$58.9	$58.9	$58.9
Out of pocket costs (millions USD)	$39.9	$34.7	$27.7	$39.9	$39.9	$39.9
Sub-total economic burden (millions USD)	$129.2	$115.1	$96.2	$121.6	$114.0	$106.3
Productivity loss due to premature deaths (millions USD)	$909.3	$909.3	$909.3	$682.0	$454.7	$227.3
Total economic burden (millions USD)	$1,038.6	$1,024.4	$1,005.6	$803.6	$568.6	$333.7

* Percentage of cholera cases hospitalized is varied under this scenario

The workdays lost are valued at a certain percentage of gross domestic product (GDP) per capita per day

For peer review only - http://bmjopen.bmj.com/site/about/guidelines.xhtml

Figures

Figure 1: Spearman's rank correlation coefficient: Economic burden of cholera in Africa

For peer review only

For peer review only - http://bmjopen.bmj.com/site/about/guidelines.xhtml

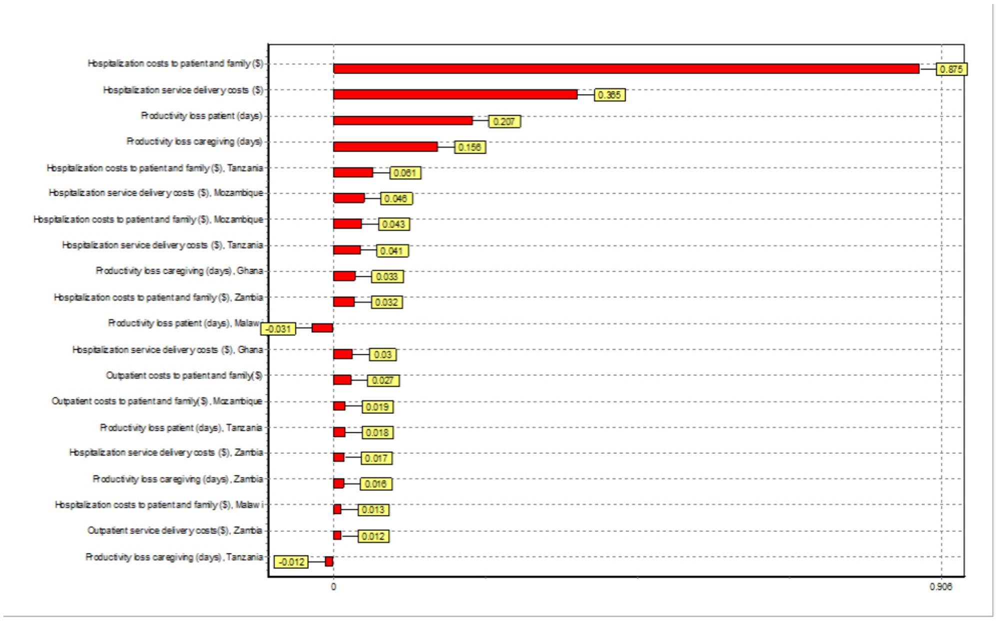

257x159mm (96 x 96 DPI)

Annex

Annex1: *Comparison of methodology and results of Kirigia et al (5) and current paper*

Parameter	Kirigia et al (5)	Current paper
Year of analysis (disease burden)	2006, 2007, 2008	2015
Year of price	2002	2015
Data source for cholera cases	WHO reported	Global burden estimate and WHO reported
Household costs	Average (non-cholera) health care costs reported in WHO survey in 16 countries	Average cholera specific household costs reported in 5 cost of illness studies
Health system costs	The hotel were assumptions based on non-cholera cases reported by WHO. The medicines and diagnostic costs were estimated based on standard practices using assumptions.	Average of reported health system costs from 5 cost of illness studies for hospitalized cases and from WHO database of outpatients. Diagnostic costs are not included.
Indirect costs	Productivity loss for the duration of illness and premature deaths were valued by gross national income per capita	Productivity loss for the duration of illness and premature deaths were valued by gross domestic product per capita
Scenario analysis	For the year (2006, 2007, 2008), Life expectancy (73, 53, 40 years)	Disease burden (3 estimates and WHO reported), Hospitalization rate (90%, 75%, 55%), GDP per capita (100%, 75%, 50%, 25%)
Range of estimated economic burden in $2015 including mortality costs	$61.4 million to $245.5 million ($39 million and $156 million in 2002 prices)	$519.3 million to $1.8 billion based on estimated cases and $29.4 million based on WHO reported cases

(5) Kirigia JM, Sambo LG, Yokouide A, Soumbey-Alley E, Muthuri LK, Kirigia DG. Economic burden of cholera in the WHO African region. BMC international health and human rights. 2009;9:8.

For peer review only

Annex 2: Economic burden of cholera in Africa by country

Country*	Population in 2015 (2)	GDP per capita US$ 2015 (18)	GDP per capita I$ 2015 (18)	Infant mortality rate per 1000 live births (28)	Life expectancy at birth 2015(21)	Current health expenditure per capita (in 2015) (29)	Cases in 2015(2)	Deaths in 2015(2)	Productivity loss due to illness (US$2015)	Public health system cost (US$2015)	Out of pocket costs (US$2015)	Economic burden excluding deaths (US$2015)	Average cost of illness (US$2015)	Productivity loss due to premature deaths (US$2015)	Total economic burden (US$2015)	Cholera economic burden per capita in US$ 2015
Angola	27,884,380	$4,355	$7,412	57.8	61.2	$108.68	19,158	728	$2,225,925	$1,132,539	$792,875	$4,151,339	$216.69	$69,296,429	$73,447,768	$2.63
Benin	10,575,962	$1,077	$2,889	64.4	60.6	$ 31.29	4,755	181	$136,614	$277,839	$196,785	$611,239	$128.55	$4,209,382	$4,820,620	$0.46
Burkina Faso	18,110,616	$576	$1,704	53.6	59.9	$33.51	6,527	248	$100,233	$381,155	$270,122	$751,509	$115.14	$3,049,953	$3,801,463	$0.21
Burundi	10,160,034	$294	$769	45.8	57.1	$14.39	10,341	393	$81,249	$602,779	$427,970	$1,111,998	$107.53	$2,341,441	$3,453,440	$0.34
Cameroon	23,298,376	$1,339	$3,469	55.7	57.6	$63.63	9,487	361	$338,811	$555,189	$392,627	$1,286,627	$135.62	$9,873,360	$11,159,987	$0.48
Cape Verde	524,740	$2,997	$6,313	18.3	72.6	$159.22	117	4	$9,356	$6,900	$4,843	$21,099	$180.30	$338,692	$359,792	$0.69
Central Africa Rep.	4,493,171	$354	$698	90.7	51.4	$21.98	5,742	218	$54,200	$335,138	$237,633	$626,971	$109.19	$1,362,506	$1,989,477	$0.44
Chad	14,110,971	$947	$2,648	76.5	52.6	$35.52	11,486	436	$290,170	$671,161	$475,372	$1,436,704	$125.08	$7,527,860	$8,964,564	$0.64
Comoros	777,435	$1,260	$2,637	56	63.5	$58.24	68	3	$2,296	$3,992	$2,827	$9,115	$133.44	$74,080	$83,195	$0.11
Congo, Democratic Republic	76,244,532	$436	$760	73.9	59.2	$20.11	136,821	5,199	$1,591,458	$7,976,946	$5,662,446	$15,230,850	$111.32	$47,788,731	$63,019,580	$0.83
Congo, Rep.	4,856,093	$2,067	$7,318	38.9	64.1	$58.45	4,770	181	$263,037	$280,720	$197,393	$741,150	$155.39	$8,569,186	$9,310,337	$1.92
Cote d'voire	23,226,148	$1,397	$3,427	64.8	53.1	$75.45	15,181	577	$565,934	$888,232	$628,278	$2,082,444	$137.17	$14,868,449	$16,950,893	$0.73
Djibouti	913,998	$2,533	$4,611	54.2	62.3	$81.73	164	5	$11,091	$9,603	$6,793	$27,487	$167.46	$295,674	$323,160	$0.35
Eritrea	3,342,818	$352	$950	34.1	64.6	$27.18	12,860	489	$120,883	$750,166	$532,229	$1,403,279	$109.12	$3,968,339	$5,371,618	$1.61
Ethiopia	100,835,453	$703	$1,811	43.8	65.0	$25.11	195,093	7,414	$3,657,892	$11,379,858	$8,074,096	$23,111,846	$118.47	$120,778,513	$143,890,360	$1.43
Gabon	1,947,690	$7,453	$17,970	36.3	65.7	$197.94	405	15	$80,456	$24,265	$16,745	$121,467	$300.20	$2,680,703	$2,802,169	$1.44
Gambia, The	2,085,860	$650	$2,375	41.9	61.0	$21.85	370	14	$6,407	$21,581	$15,302	$43,290	$117.08	$198,500	$241,790	$0.12
Ghana	27,849,203	$1,754	$5,524	38.9	62.4	$82.41	6,794	258	$383,782	$439,711	$524,804	$1,348,298	$198.45	$10,087,937	$11,436,235	$0.41
Guinea	11,432,096	$712	$1,838	68.5	59.4	$42.25	5,656	215	$107,438	$329,968	$234,059	$671,465	$118.73	$3,239,028	$3,910,493	$0.34
Guniea-Bissau	1,737,207	$644	$1,679	59.5	57.0	$ 38.14	1,142	43	$19,619	$66,610	$47,263	$133,492	$116.89	$559,084	$692,575	$0.40
Kenya	47,878,339	$1,453	$3,235	33.3	66.7	$66.02	67,091	2,549	$2,601,329	$3,920,223	$2,776,614	$9,298,166	$138.59	$87,849,671	$97,147,837	$2.03

For peer review only

Lesotho	2,059,011	$1,167	$3,174	71.2	53.7	$97.47	1,768	67	$55,025	$103,310	$73,170	$231,505	$130.94	$1,466,453	$1,697,959	$0.82
Liberia	4,472,229	$764	$1,421	58.5	62.0	$71.94	2,137	81	$43,582	$124,613	$88,441	$256,636	$120.09	$1,370,686	$1,627,323	$0.36
Madagascar	24,234,080	$400	$1,470	40.8	65.5	$22.13	22,766	865	$243,165	$1,328,860	$942,189	$2,514,214	$110.44	$8,084,510	$10,598,724	$0.44
Malawi	16,745,305	$354	$1,129	40.3	62.7	$34.00	10,209	388	$118,651	$718,229	$267,074	$1,103,954	$108.14	$3,068,258	$4,172,211	$0.25
Mali	17,438,772	$773	$2,115	67.2	57.5	$30.83	10,070	383	$207,579	$588,249	$416,755	$1,212,582	$120.42	$6,033,505	$7,246,088	$0.42
Mauritania	4,046,304	$1,303	$4,297	55.4	63.1	$53.61	3,609	137	$125,505	$211,067	$149,361	$485,933	$134.64	$4,021,206	$4,507,139	$1.11
Mozambique	27,042,001	$529	$1,195	59.3	57.7	$27.38	50,811	1,931	$655,324	$2,409,795	$1,875,380	$4,940,499	$97.23	$20,923,973	$25,864,472	$0.96
Namibia	2,314,901	$5,160	$11,320	33.5	63.8	$466.11	610	23	$83,980	$36,017	$25,245	$145,241	$238.10	$2,701,262	$2,846,503	$1.23
Niger	20,001,663	$363	$964	52	59.7	$25.72	16,212	616	$156,885	$945,879	$670,946	$1,773,710	$109.41	$4,750,822	$6,524,533	$0.33
Nigeria	181,137,454	$2,726	$6,053	78.7	53.0	$97.52	134,155	5,098	$9,758,067	$7,856,671	$5,552,111	$23,166,849	$172.69	$255,879,611	$279,046,459	$1.54
Rwanda	11,369,066	$736	$1,907	30.8	66.7	$46.40	15,171	577	$298,052	$885,490	$627,864	$1,811,406	$119.40	$10,076,243	$11,887,649	$1.05
São Tomé and Principe	199,439	$1,570	$3,033	27	66.5	$86.91	39	1	$1,633	$2,282	$1,614	$5,529	$141.78	$37,118	$42,647	$0.21
Senegal	14,578,450	$1,187	$3,118	35.1	66.8	$50.96	7,252	276	$229,646	$424,220	$300,130	$953,996	$131.55	$7,777,910	$8,731,906	$0.60
Sierra Leone	7,171,909	$600	$1,433	87.8	51.4	$119.82	5,177	197	$82,813	$302,022	$214,254	$599,089	$115.72	$2,085,448	$2,684,538	$0.37
Somalia	13,797,204	$293	$775	83.1	55.9	NA	11,220	359	$87,847	$656,748	$464,349	$1,208,944	$107.75	$2,076,914	$3,285,858	$0.24
South Sudan	10,715,657	$1,225	$2,002	63.7	56.3	$28.05	17,098	650	$558,887	$1,000,988	$707,614	$2,267,490	$132.62	$15,851,280	$18,118,769	$1.69
Sudan	38,902,948	$1,698	$4,282	45.4	64.3	$158.02	26,311	842	$1,191,987	$1,540,819	$1,088,902	$3,821,708	$145.25	$32,772,322	$36,594,030	$0.94
Swaziland	1,104,038	$3,780	$10,038	47.9	57.1	$231.42	1,384	53	$139,559	$81,285	$57,278	$278,122	$200.96	$4,053,661	$4,331,783	$3.92
Tanzania	51,482,638	$948	$2,782	41.9	65.0	$32.09	84,550	3,213	$1,945,796	$5,254,900	$2,174,112	$9,374,807	$110.88	$70,515,725	$79,890,533	$1.55
Togo	7,323,162	$564	$1,546	51.4	59.9	$35.21	4,919	187	$73,986	$287,293	$203,577	$564,855	$114.83	$2,253,130	$2,817,985	$0.38
Uganda	38,225,447	$647	$2,272	38.6	59.6	$39.77	38,066	1,446	$656,973	$2,222,025	$1,575,392	$4,454,389	$117.02	$19,855,976	$24,310,365	$0.64
Zambia	15,879,370	$1,310	$3,844	44.5	61.4	$58.52	20,618	784	$749,934	$1,203,369	$440,605	$2,393,908	$116.11	$22,512,900	$24,906,808	$1.57
Zimbabwe	13,814,642	$1,425	$2,640	38.5	60.4	$94.29	10,462	398	$397,751	$612,289	$432,978	$1,443,018	$137.93	$12,217,700	$13,660,718	$0.99
Total/average	936,340,81			52.26	60.61		1,008,642	38,104	$30,510,80	$58,850,99	$39,866,41	$129,228,2		$909,344,1	$1,038,572,	$39.20

For peer review only

	2								7	3	8	18		33	351	

For peer review only

Model-based estimation of the economic burden of Cholera in Africa

Journal:	*BMJ Open*
Manuscript ID	bmjopen-2020-044615.R1
Article Type:	Original research
Date Submitted by the Author:	13-Feb-2021
Complete List of Authors:	Mogasale, Vittal; International Vaccine Institute, Policy and Economic Research<br>Ngogoyo, Samuel ; County government of Nyandarua, Department of Health<br>Mogasale, Vijayalaxmi; Yenepoya Medical College Hospital, Department of Pediatrics
**Primary Subject Heading**:	Global health
Secondary Subject Heading:	Health economics, Health policy, Infectious diseases
Keywords:	Health economics < HEALTH SERVICES ADMINISTRATION & MANAGEMENT, Health policy < HEALTH SERVICES ADMINISTRATION & MANAGEMENT, Public health < INFECTIOUS DISEASES, Tropical medicine < INFECTIOUS DISEASES

SCHOLARONE™ Manuscripts

I, the Submitting Author has the right to grant and does grant on behalf of all authors of the Work (as defined in the below author licence), an exclusive licence and/or a non-exclusive licence for contributions from authors who are: i) UK Crown employees; ii) where BMJ has agreed a CC-BY licence shall apply, and/or iii) in accordance with the terms applicable for US Federal Government officers or employees acting as part of their official duties; on a worldwide, perpetual, irrevocable, royalty-free basis to BMJ Publishing Group Ltd ("BMJ") its licensees and where the relevant Journal is co-owned by BMJ to the co-owners of the Journal, to publish the Work in this journal and any other BMJ products and to exploit all rights, as set out in our licence.

The Submitting Author accepts and understands that any supply made under these terms is made by BMJ to the Submitting Author unless you are acting as an employee on behalf of your employer or a postgraduate student of an affiliated institution which is paying any applicable article publishing charge ("APC") for Open Access articles. Where the Submitting Author wishes to make the Work available on an Open Access basis (and intends to pay the relevant APC), the terms of reuse of such Open Access shall be governed by a Creative Commons licence – details of these licences and which Creative Commons licence will apply to this Work are set out in our licence referred to above.

Other than as permitted in any relevant BMJ Author's Self Archiving Policies, I confirm this Work has not been accepted for publication elsewhere, is not being considered for publication elsewhere and does not duplicate material already published. I confirm all authors consent to publication of this Work and authorise the granting of this licence.

For peer review only

Model-based estimation of the economic burden of Cholera in Africa

*Vittal Mogasale [a] *, Samuel Mwaura Ngogoyo [b], Vijayalaxmi V Mogasale [c]*

[a] Policy and Economic Research Department, International Vaccine Institute, Seoul, South Korea.

[b] County Government of Nyandarua. Department of Health, Ol Kalou, Kenya.

[c] Department of Pediatrics, Yenepoya Medical College and Research Center, Mangalore, India

***Corresponding author:**

Dr. Vittal Mogasale, Policy and Economic Research Department, International Vaccine Institute, SNU Research Park, 1 Gwanak-ro, Gwanak-gu Seoul 151-742. South Korea. Tel: +82 2 881 1379(Dir); +82 2872 2801 (Ext 379); Fax: + 82 2 872 2803

E-mail: vmogasale@ivi.int; vmogasale@gmail.com

For peer review only

Abstract

Objectives

To estimate the economic burden of cholera in Africa.

Settings

Cholera affected 44 countries in Africa.

Participants

The analysis used data from public sources in Africa published until September 2019.

Methods

Based on existing data from field-based cost-of-illness studies, estimated cholera incidence rates, and reported cholera cases to WHO, this research estimates the economic burden of cholera in Africa from a societal perspective with 2015 as the base year. The estimate included out-of-pocket costs, public health system costs, productivity loss related to illness, and an optional productivity loss related to premature deaths valued by the human capital approach. As various input data such as cholera incidence, hospitalization rates, and the number of workdays lost were not well defined, a series of scenario analyses and uncertainty analyses, accounting for unknowns and data variability, was conducted. Similarly, the value of time lost due to illness and deaths using the human capital approach was explored through scenario analyses.

Results

In 2015, an estimated 1,008,642 cases in 44 African countries resulted in an economic burden of US$130 million from cholera-related illness and its treatment. When the estimated 38,104 cholera deaths were included in the analysis, the economic burden increased to US$1 billion or international $2.4 billion for the same year. At the same time, when only the 71,126 cases and 937 deaths reported to the WHO are considered, the economic burden was only US$68 million for the year 2015. The estimates of economic burden are thus heavily dependent on the cholera incidence rate, how time lost due to illness and deaths are calculated, hospitalization rates, and hospitalization costs.

Conclusion

For peer review only - http://bmjopen.bmj.com/site/about/guidelines.xhtml

The findings can be used as an economic justification for cholera control in Africa and for generating value-for-money evidence to underpin *Ending Cholera – A Global Roadmap to 2030* with considerations to study limitations.

Strengths and limitations of this study

- Model-based estimation of the economic burden of cholera in 44 African countries was conducted using various secondary data sources.
- The average inpatient health system costs, out of pocket expenses, and the number of days of productivity lost related to cholera treatment from five African papers that presented data was directly applied to countries that did not have data.
- The estimated number of cholera cases was derived from the global burden of disease study and the World Health Organization report.
- The productivity loss related to cholera illness and premature deaths were valued by the human capital approach using gross domestic product per capita.
- Cholera incidence rates, cholera hospitalization rates, and cholera hospitalization costs are the most uncertain parameters that drove the results.

Key Words

cholera, economic burden, Africa, costs, cholera elimination

For peer review only - http://bmjopen.bmj.com/site/about/guidelines.xhtml

Introduction

Cholera, an acute, diarrheal illness caused by infection of the intestine with the toxigenic bacterium *Vibrio cholerae* is linked to poor water, sanitation, and hygiene (WASH) conditions (1). In cholera-endemic countries, an estimated 2.8 million cases and 91,000 deaths occur annually, due to cholera (2). However, the actual number of reported cases to the World Health Organization (WHO) is significantly lower. Globally, around 132,000 cases and 2400 deaths were reported to the WHO in 2016 (3). Cumulatively, from 2000 to 2016, 3.4 million cholera cases and 65,000 deaths worldwide were reported to WHO (3). Countries in Africa continue to experience a disproportionately high number of cholera outbreaks resulting in a high burden of disease and deaths. In 2016, seventeen countries in Africa accounted for 71,000 cases (54% of global cases) with 1,760 deaths (42%of deaths globally) (3). Furthermore, the average case fatality rate of 2.5% in African countries is substantially higher than the global average of 1.8% (3).

When measured as an economic burden, cholera has a significant economic impact on health systems and individuals. The societal cost per cholera case is believed to be more than $1,000 when the wider socio-economic costs such as cost to the health system, the patient's family, and income loss due to death, are taken into account (4). While several studies have been done in various countries on the economic burden of cholera, the cost-effectiveness of interventions, and other related areas of interest, there is a lack of any recent studies estimating the economic burden in Africa looking into the areas of loss of productivity, hospitalization and outpatient costs, quantified at the national and regional levels. A model-based estimate in 2007 reported an annual economic loss ranging from US$39 million to US$64.2 million as a result of cholera in $2002 prices and based on regional life expectancies ranging from 40 to 73 years in Africa (5). The study used several cost assumptions around standard treatment and diagnostic practices which may not be the current situation in Africa and may be outdated.

A recent economic burden estimate in Asia used the actual estimated cost of illness collected from countries to project the economic burden while using a variety of scenario and sensitivity analyses to demonstrate the uncertainties around the estimated and reported cholera cases to WHO (6). This kind of economic burden estimate provides a better understanding of economic offsets that can be achieved through cholera control measures and can be used as evidence to make a case for the effectiveness of cholera control. We used the same methodological approach as in Asia (6) and used the latest data available from field settings in Africa to quantify the economic burden of cholera in Africa.

For peer review only - http://bmjopen.bmj.com/site/about/guidelines.xhtml

Methods

The research involved the selection of countries, identification of cost-of-illness studies to define out-of-pocket expenses, productivity loss, and health system costs to estimate and extrapolate the economic burden, and finally, conduct sensitivity analysis and scenario analyses. Patients or the public were not involved in the design, or conduct, or reporting, or dissemination plans of this research.

Conceptual framework

The primary input needed for estimating the economic burden is the cost-of-illness studies conducted around cholera cases at health facilities in African countries. Such studies provide three sets of key information (1). Out-of-pocket (OOP) expenditures borne by patients and families for cholera treatment which could include medical costs such as medicines and intravenous fluids, or non-medical costs expenditures on transport and food (2). Loss of income, which is often referred to as productivity costs related to illness, because the patient is unable to work and someone needs to take care of the patient. This is often presented in the number of workdays lost (3). Cost to the health system for providing the cholera treatment which includes costs related to staff, provision of services, maintenance of hospital facilities, etc.

The cost-of-illness data is typically not available from most African countries. In such situations, cost-of-illness was extrapolated to African countries using available data from field studies in the region (out of pocket expenses, inpatients costs, and productivity loss) and WHO estimates (outpatient costs) such that each country has a unit cost of illness (cost of illness per cholera case). When the total number of cholera cases is multiplied by the unit cost-of-illness, the economic burden for that country is derived which is summated at the regional level. However, all the input costs and the number of cases are not constant numbers. There may be several biases and reported variations such as under-reporting or over-estimations because of which we conduct a series of scenario analyses and uncertainty analyses as described below so that the limitations and boundaries are well understood.

Country selection and disease burden estimation

African countries as defined by the United Nations (7), that either reported cholera cases to WHO or were estimated to have cholera cases, were selected for the analysis. The year 2015 was selected as the reference year for data comparability purposes since both, disease burden estimates (2) and cases reported to WHO (8), were available for that year. Most often than not, cholera is under-reported by many countries because of reasons such as the lack of diagnostics and reagents to test cholera, inadequacies in the surveillance system, differences in case definition, and the reluctance of authorities to acknowledge or report cholera (9). Therefore, the data in the WHO report is under-reported and an economic burden

For peer review only - http://bmjopen.bmj.com/site/about/guidelines.xhtml

estimate based on the WHO report will be an underestimation. Whereas the 2015 global burden of disease estimate (2) has considered several incidence scenarios as part of sensitivity analysis, of which three were used in this analysis. In total, four cholera incidence scenarios were used in the analysis as listed below.

(a) Base case: The Global Burden of Cholera study (2) had extrapolated the cholera incidence data from Beira, Mozambique (10) to the rest of Africa after applying the incidence rate to the population at risk based on the percentage of people without sustainable access to improved water. We used the same incidence numbers in the base case. This methodology assumes that only a part of the population is at risk for cholera. This was one of the sensitivity analyses in the Global Burden of Cholera study (2).

(b) Liberal case: The incidence rate from Beira, Mozambique was extrapolated to the rest of Africa after applying an incidence correction assumption by African WHO mortality strata D (AFR-D) (11). It was assumed that AFR-D had half the incidence as Beira, while the rest of African countries were assumed to have the same incidence rate as Beira (2). Here the assumption is that the whole of the population is at risk for cholera, but with different incidence rates. This was the base case in the Global Burden of Cholera publication (2).

(c) Conservative case: As other sensitivity analyses used in the 2015 global burden of disease estimate produced a similar number of cholera cases in Africa, a conservative estimate was derived by applying half of the incidence rate as the base case.

(d) WHO report case: A separate scenario analysis was conducted based on the number of cholera cases and deaths reported to WHO in 2015.

The 2015 global burden of disease study (2) had applied a 3.8% case fatality rate to estimate death, and the same was applied in the first three scenario analyses.

Cholera cost-of-illness

As described previously in the conceptual framework, there are three types of costs: out-of-pocket costs, productivity loss, and health system costs. These costs were extracted from literature based on actual studies conducted in healthcare settings. A previous systematic literature review (4) had identified two cost-of-illness studies conducted in Africa, one in Mozambique (12) and another in Tanzania (13). Furthermore, a PubMed search in September 2019 yielded three more studies published subsequently --

For peer review only - http://bmjopen.bmj.com/site/about/guidelines.xhtml

one each from Malawi (14), Ghana (15), and Zambia (16). Data from these 5 studies were extracted and used as inputs in the analysis.

Out-of-Pocket (OOP) costs

The OOP costs for hospitalized cases were reported for all five papers used in the study, while only Malawi reported the OOP costs for outpatient cases. There are two types of OOP costs, direct medical, and direct non-medical costs. Direct medical costs include expenditure accrued by patients for diagnosis, medicines, and other costs directly related to the treatment of cholera. Direct non-medical costs include expenditures on travel to the healthcare facility, room and boarding, food, and other costs not directly related to treatment. All the costs reported in the different years were converted to 2015 US dollars by adjusting them based on annual percent changes in world consumer prices as reported by the International Monetary Fund (IMF) (17). For countries that did not report OOP costs either for hospitalization or outpatient, the average cost derived from the rest of the reported costs were applied directly.

Lost productivity

Loss of productivity due to illness was estimated based on the average number of lost workdays for patients and caregivers throughout the illness and the recuperation period documented separately for patients and caregivers. Lost workdays for Ghana, Malawi, and Mozambique were extracted directly from their respective sources (12, 14, 15). Data from Tanzania only includes the duration of illness and does not account for the loss of productivity during the recuperation period (13). While the study in Zambia did not give an exact average, we were able to estimate the average based on the survey results (16). The ranges of the lost days were calculated based on the range derived from the study in Tanzania, where the variability of 20% was observed. The lost days were then multiplied by the average gross domestic productivity (GDP) per capita (18) to derive total productivity loss per cholera episode. As cholera incidence may be higher in people with low income, we used three scenarios that estimated productivity loss at 75%, 50%, and 25% of GDP per capita.

Health system costs

Service delivery costs were also extracted from the papers for each hospitalized and outpatient cases. Health facility costs for service delivery included personnel, medicines, diagnostic tools, medical equipment, infrastructure, beds, and utilities that patients are not responsible for paying. Of the five publications used in this study, Malawi, Tanzania, and Mozambique reported health service delivery costs for hospitalized cases. For countries without reported costs, the mean hospitalization health facility costs for these three African countries were applied. Only Malawi reported outpatient service delivery costs.

For peer review only - http://bmjopen.bmj.com/site/about/guidelines.xhtml

The country-specific average outpatient costs for health centers with and without beds from the WHO-CHOICE (19) project were applied to all other countries.

Loss of productivity because of death

To estimate the productivity loss due to premature death from cholera, the mean age of cholera death was derived from a multi-country cholera surveillance project that reported 873 deaths in six African surveillance zones (20). Life expectancy at birth for each country was subsequently subtracted by the mean age of cholera. Information for life expectancy used the 2015 data provided by the World Bank (21). To convert the years lost to monetary values, GDP per capita (18) was multiplied by the number of deaths from cholera, and the number of productive years lost per death. A discount rate of 3% was applied to future earning potential. The GDP per capita although may be considered an overestimate considering cholera may disproportionately affect poor people, it allows accounting for everyone affected equally and their variable life expectancy.

Hospitalization rate

Only the study conducted in Malawi reported a hospitalization rate, at 90% hospitalized and 10% receiving care for less than 12 hours, which is analogous to outpatient care. Other studies included in the analysis were unclear whether all cases were hospitalized, or only hospitalized cases were accounted for. Since 90 to 100% hospitalization is considered high, the analysis was conducted based on three scenarios: (a) under the assumption of 90% hospitalization, (b) average of 75% hospitalization, and (c) average of 55% hospitalization. Assumption (b) was determined based on a previous investment case study (22, 23) while assumption (c) was based on results from an economic burden study conducted for Asian countries (6) which is the average hospitalization rate in Asia.

Probabilistic multi-variate sensitivity analysis

A probabilistic multi-variate sensitivity analysis was conducted using Ersatz (24). A beta-PERT distribution was used for cost inputs which use the minimum, mean, and maximum as input parameters. The beta-PERT distribution is most suited for cost modeling or parameters obtained through the survey that present minimum, a maximum, and most likely values (24). To estimate 95% confidence intervals a Monte Carlo simulation was conducted based on 5,000 random draws for each input parameter based on the distribution allocated.

Scenario Analysis

For peer review only - http://bmjopen.bmj.com/site/about/guidelines.xhtml

Among all input parameters, disease burden (the number of cases) and hospitalization rates had limited information that could be captured in one single number with uncertainty ranges. Therefore, scenario analyses were conducted for both disease burden and hospitalization rates. Disease burden scenarios included three estimates as explained earlier and is based on a previous publication (2) and the reported cholera cases and deaths to WHO (8). The hospitalization rate had three scenarios as described above namely 90%, 75%, and 55% hospitalization rates. As productivity loss is driving overall costs, we conducted scenario analysis by valuing loss of workdays at 75%, 50%, and 25% of GDP per capita.

All the costs were converted to their equivalent value in 2015 US dollars (US$). For the conversion, the annual percent changes in world consumer prices as established by the IMF were applied (17). The results were presented both in 2019 US$ and 2019 International dollars (I$). For the conversion from US$ to I$, a country-specific multiplication factor from World Bank data that ranged from 1.63 to 3.67 was used (18).

The comparison of methodology with previous economic burden study

The previous cholera economic burden study estimated the costs in 2002 prices for WHO reported cholera cases in 2005, 2006, and 2007 (5). It used WHO data sources and assumptions based on standard clinical practices for estimating health system (hotel cost, diagnostic costs, and medicine costs) and out of pocket (household) costs. A methodological comparison table is provided in **Annex 1**. The current study estimated cholera economic burden in Africa using available data from the field-based cost of illness study and most recent disease burden estimates available and in a comparable approach to economic burden estimate in Asia (6).

Results

Based on a literature review, average workday loss of 5.76 (range 2.96 to 9.60) and 3.86 (range 3.09 to 4.64) was estimated for cholera cases and caregivers respectively, irrespective of their hospitalization status **(Table 1)**. The OOP costs were US$ 38.87 (range $6.07 to $109.94) for hospitalized cases and US$ 5.71 (range $0.89 to $16.15) for those who visited the health facility but did not get hospitalized. The health facility costs were estimated at US$ 65.77 and US$ 2.84 for hospitalized and outpatient cases respectively. The mean age of death due to cholera was 23.74 years. The analysis included 44 African countries and the list of countries included in the analysis is available in **Annex 2**.

In the base case analysis, 1,008,642 cases of all ages were included from 44 African countries, while 71,176 cholera cases from 16 countries were reported to WHO (**Annex 2**). The number of deaths

For peer review only - http://bmjopen.bmj.com/site/about/guidelines.xhtml

estimated in the base case was 38,104 from 44 countries, while 937 were reported to WHO. Excluding deaths, the total economic burden is US$130 million and I$ 315 million for the year 2015 (**Table 2**). When productivity losses related to deaths are taken into account, the total economic burden increases to US$1 billion, and I$2.4 billion for the same year. Country-specific economic burden and unit costs are provided in **Annex 2** which shows the per capita economic burden of cholera ranges from US$0.1 to US$3.9 with an average of US$1.1.

Scenario analyses

The scenario analyses showed that when assumptions on the number of cholera cases changed, the economic burden excluding death ranged from $64.6 million to $227.2 million. The estimated/ reported number of cases influences the economic burden considerably and drops to $8.6 million when WHO reported cholera cases are considered in the analysis (**Table 3**).

The scenario analysis that varied hospitalization rates had a relatively small impact on the overall economic burden. When workdays lost are valued at 75%, 50%, and 25% of GDP per capita, a large reduction of economic burden was observed (**Table 4**).

Probabilistic multi-variate sensitivity analysis

Probabilistic multi-variate sensitivity analysis showed OOP costs during hospitalization, service delivery costs at hospitalization, and the number of workdays lost among cholera patients and caregivers, have the largest impact on the results (**Figure 1**).

Discussion

The total economic burden of cholera in Africa was estimated at US$1 billion (I$2.5 billion) which was around US$1.1 per capita for the year 2015, of which only US$0.06 is borne by the public health system. Lost productivity due to premature death due to cholera was estimated to be US$900 (I$2.2 billion) for the year 2015. Productivity loss due to premature death accounted for 87.7% of the total economic burden of cholera in Africa. When costs linked to productivity loss are excluded, 45.6% of the costs were borne by the health system, with the rest borne by affected families including 23.6% as OOP costs and 30.8% as productivity loss due to inability to work. Probabilistic multi-variate sensitivity analysis demonstrated that

For peer review only - http://bmjopen.bmj.com/site/about/guidelines.xhtml

For peer review only

the costs around hospitalization and loss of workdays are driving the overall costs, which the scenario analysis showed that the economic burden is heavily dependent on how we value loss of workdays.

The Global Task Force for Cholera Control (GTFCC) has proposed an ambitious plan to reduce 90% of cholera cases by 2030 as a part of the *Ending Cholera- a Global Roadmap to 2030* (25). This plan needs substantial investment for cholera control including in safe water, sanitation and hygiene, and oral cholera vaccines, in addition to the mapping of cholera hotspots, surveillance, treatment, and community engagement. This economic burden estimate may help in expanding thinking beyond a governmental perspective to a wider societal perspective, and whereby many of the African countries may recognize the value of making such investments in cholera control. The economic burden of cholera as estimated here has comparative value for decision-makers on the quantum of investment and its relative return and is also useful for conducting economic evaluation and quantifying the value of such investments.

A previous study on the economic burden of cholera in Africa showed costs of US$39 million to US$156 million, depending on the assumptions on life expectancy (73, 53, 40 years) and year of reported cholera cases (2005, 2006, and 2007) (5). When converted into 2015 US$ by adjusting for world inflation, the economic burden ranged from $61.4 million to $245.5 million. Whereas the current study estimated an economic burden of $519.3 million to $1.8 billion based on estimated cholera cases and $29.4 million based on WHO reported cases in 2015. This was a model-based study and had used standard assumptions. While the current study tries to extrapolate field-based data to estimate the economic burden to around 1 billion USD in 2015. Although this study tries to draw inputs from field data, such data has limitations. The biggest driver for the cost difference is the incidence number of cholera cases and deaths. The previous study used cholera incidence deaths as per WHO reports, while the current study uses a comparative scenario analysis (Table 2). When WHO reports are used for incidence estimates, the economic burden in the current study is US$29.4 million which is low compared to the previous publication. This is because a larger number of cases and deaths were reported to WHO in 2005-2007 compared to 2015. The number of cases ranged from 125,018 to 203,564 and deaths ranged from 2,230 to 5,281 in 2005-2007 while it was only 71,16 cases and 736 deaths in 2015.

The economic burden of cholera estimation study in Asia (6) that had used a comparative methodology based on field data had shown an economic burden of US $987.1 for 2015, which is comparable to the US$ 1 billion reported here for Africa. However, the number of cholera cases is estimated to be lower in Africa compared to Asia in the base-case (1,008,642 vs 851,396), indicating a relatively higher cost per case in Asia. The average unit cost per case in Asia was US$1,159 compared to US$1,029 in Africa.

For peer review only - http://bmjopen.bmj.com/site/about/guidelines.xhtml

Limitations

This estimate of the economic burden of cholera used the latest available field-based information in its analysis, but not without limitations.

Firstly, the incidence data on cholera were estimated previously (2) by extrapolating field-based data; however, these were not real numbers but estimations. While, we also used WHO reported cases and deaths in one scenario which showed a substantially lower economic burden, but even these cannot be considered as real numbers as the WHO reported cases are known to be an underestimate. With cholera incidence numbers being the main cost drivers in this analysis, the potential unknowns in the incidence data and resultant variations in economic burden need to be considered when using the results. A scenario analysis that varied incidence cases and deaths are presented to understand this limitation.

Secondly, the entire cost-of-illness field data inputs came from five African papers, four of which had only costs for hospitalized cases which means that the cost-of-illness data is heavily dependent on either extrapolation or assumptions. It is known that in many African countries cholera treatments are provided through specific centers known as 'cholera treatment centers' often set up adjacent to existing health facilities, close to cholera-affected communities. Most severe cases are hospitalized in these centers, while others are kept under observation along with the provision of rehydration and treatment. This situation creates a challenge in differentiating the typical categorization of cholera cases as hospitalized and outpatient cases, with most studies resulting in reporting everyone as hospitalized. The health facility costs of such specialized treatment centers could be different from typical facility-based service provision. In the absence of outpatient data, we have used WHO-CHOICE data that may not represent typical cholera treatment situations. Because the base-case assumes 90% of cholera cases are hospitalized, this bias is minimized. In addition, a scenario analyses with a range of hospitalization rates was presented.

Thirdly, OOP costs and the number of workdays lost are collected from patient and family surveys which are subject to many biases. The OOP costs are different in different countries, and range depending upon the patient's location, type of health facility, travel time and distance, and the health care system. It is also subject to the limitations of surveys such as how and when the data was collected. This data was extrapolated to all African countries from 5 study sites. The extrapolation methods may have resulted in underestimation or overestimation of OOP costs, the direction of which is difficult to ascertain. Extensive sensitivity analyses are conducted to understand these limitations.

For peer review only - http://bmjopen.bmj.com/site/about/guidelines.xhtml

Fourthly, the overall economic burden is heavily dependent on how the lost workdays and years lost due to death are valued. Cholera is known to affect economically marginalized people more often, and a GDP per capita estimation of valuing their lost time could be an overestimation. Also, some of the people may not be in the workforce, may not earn in the future, but still, their time is valued. If an ill worker is replaced, it may affect family income but may not affect the overall economy. Thus, the potential income loss presented in this analysis need not represent the true income loss in African countries. To understand these limitations a scenario analysis where lost workdays and death years are valued at 75%, 50%, and 25% of GDP per capita.

Lastly, cholera is known to affect the tourism and export industries which are not accounted for here, resulting in an underestimation of the economic burden. Similarly, the costs of outbreak control are not included here. Also, health facility costs do not include diagnostic costs.

Conclusion

This study estimates the economic burden of cholera in 44 African countries, which can be used as evidence for making the case for cholera control measures such as investments in improving water, sanitation, and hygiene, a better surveillance system, the use of oral cholera vaccination, and other measures described in the *Ending Cholera- a Global Roadmap to 2030* (25). There is some evidence on the cost-effectiveness of using oral cholera vaccines in the control of cholera (4). This data can serve as input for conducting economic evaluation such as a cost-effectiveness analysis of using oral cholera vaccine in the *Ending Cholera- a Global Roadmap to 2030,* with additional information such as vaccine delivery costs (26) and vaccination campaigns (27). Study limitations, which include uncertainty related to cholera incidence rates, cholera hospitalization rates, valuation of lost time, and hospitalization costs, need to be thoroughly considered before using the data for calculating the economic burden of cholera in Africa.

Contribution

VM developed the concept, conducted the final analysis, and wrote the manuscript. SMN assisted in data collection and conducted the initial analysis. VVM assisted in data analysis and writing the manuscript. All authors have reviewed and accepted the final manuscript.

Funding

For peer review only - http://bmjopen.bmj.com/site/about/guidelines.xhtml

This research was conducted by International Vaccine Institute and supported by the Swedish International Development Cooperation Agency [5410054]; the Korea International Cooperation Agency (KOICA)[KOICA-IVI 6/16/2017]; the UK Foreign, Commonwealth, and Development Office, and Wellcome [214662/Z/18/Z]; and Bill & Melinda Gates Foundation [OPP38590]. The International Vaccine Institute acknowledges its donors including the Republic of Korea and the Republic of India.

Acknowledgment

The authors thank Ms. Monica Moon for initial support in data collection and analysis, Mr. Satyajit Sakar for editing support.

Patient and public involvement

Patients and/or the public were not involved in the design, or conduct, or reporting, or dissemination of this research.

Competing interest statement*None declared*

Data sharing statement

All data are presented in the manuscript, no additional data available

References

1. WHO. Cholera. 2020 [cited 2020 April 12]. Available from: https://www.who.int/news-room/fact-sheets/detail/cholera.
2. Ali M, Nelson AR, Lopez AL, Sack DA. Updated global burden of cholera in endemic countries. PLoS Negl Trop Dis. 2015;9(6):e0003832.
3. Cholera, 2016. Releve epidemiologique hebdomadaire / Section d'hygiene du Secretariat de la Societe des Nations = Weekly epidemiological record / Health Section of the Secretariat of the League of Nations. 2017;92(36):521-30.
4. Hsiao A, Hall AH, Mogasale V, Quentin W. The health economics of cholera: A systematic review. Vaccine. 2018;36(30):4404-24.
5. Kirigia JM, Sambo LG, Yokouide A, Soumbey-Alley E, Muthuri LK, Kirigia DG. Economic burden of cholera in the WHO African region. BMC international health and human rights. 2009;9:8.
6. Mogasale V, Mogasale VV, Hsiao A. Economic burden of cholera in Asia. Vaccine. 2020;38 Suppl 1:A160-A6.
7. UN. Composition of macro geographical (continental) regions, geographical sub-regions, and selected economic and other groupings; Geographical region and composition2011 February 02, 2014 [cited 2014 February 02]. Available from: http://unstats.un.org/unsd/methods/m49/m49regin.htm.
8. Cholera, 2015. Releve epidemiologique hebdomadaire / Section d'hygiene du Secretariat de la Societe des Nations = Weekly epidemiological record / Health Section of the Secretariat of the League of Nations. 2016;91(38):433-40.

For peer review only - http://bmjopen.bmj.com/site/about/guidelines.xhtml

9. Ganesan D, Gupta SS, Legros D. Cholera surveillance and estimation of burden of cholera. Vaccine. 2020;38 Suppl 1:A13-A7.
10. Lucas ME, Deen JL, von Seidlein L, Wang XY, Ampuero J, Puri M, et al. Effectiveness of mass oral cholera vaccination in Beira, Mozambique. N Engl J Med. 2005;352(8):757-67.
11. Ali M, Lopez AL, You YA, Kim YE, Sah B, Maskery B, et al. The global burden of cholera. Bull World Health Organ. 2012;90(3):209-18A.
12. Poulos C, Riewpaiboon A, Stewart JF, Clemens J, Guh S, Agtini M, et al. Costs of illness due to endemic cholera. Epidemiology and infection. 2012;140(3):500-9.
13. Schaetti C, Weiss MG, Ali SM, Chaignat CL, Khatib AM, Reyburn R, et al. Costs of illness due to cholera, costs of immunization and cost-effectiveness of an oral cholera mass vaccination campaign in Zanzibar. PLoS Negl Trop Dis. 2012;6(10):e1844.
14. Ilboudo PG, Huang XX, Ngwira B, Mwanyungwe A, Mogasale V, Mengel MA, et al. Cost-of-illness of cholera to households and health facilities in rural Malawi. PloS one. 2017;12(9):e0185041.
15. Awalime DK, Davies-Teye BBK, Vanotoo LA, Owoo NS, Nketiah-Amponsah E. Economic evaluation of 2014 cholera outbreak in Ghana: a household cost analysis. Health Econ Rev. 2017;7(1):45.
16. Tembo T, Simuyandi M, Chiyenu K, Sharma A, Chilyabanyama ON, Mbwili-Muleya C, et al. Evaluating the costs of cholera illness and cost-effectiveness of a single dose oral vaccination campaign in Lusaka, Zambia. PloS one. 2019;14(5):e0215972.
17. IMF. Inflation rate, average consumer prices 2020 [cited 2020 26 April]. Available from: https://www.imf.org/external/datamapper/PCPIPCH@WEO/OEMDC/ADVEC/WEOWORLD.
18. WorldBank. The World Bank data on GDP 2017 [Available from: http://data.worldbank.org/indicator/NY.GDP.MKTP.CD.
19. WHO-CHOICE. Country-specific unit costs. Geneva: CHOosing Interventions that are Cost Effective (WHO-CHOICE), World Health Organization; 2008 [cited 2012 5/01/2012]. Available from: http://www.who.int/choice/country/country_specific/en/index.html.
20. Sauvageot D, Njanpop-Lafourcade BM, Akilimali L, Anne JC, Bidjada P, Bompangue D, et al. Cholera Incidence and Mortality in Sub-Saharan African Sites during Multi-country Surveillance. PLoS Negl Trop Dis. 2016;10(5):e0004679.
21. WorldBank. Life expectancy at birth, total (years) 2017 [cited 2017 25 April]. Available from: http://data.worldbank.org/indicator/SP.DYN.LE00.IN.
22. Ann Levin DD, YE Kim, J Clemens, AL Lopez, M Ali, C Burgess, S Shin, T Wierzba. An Investment Case for the Accelerated Introduction of Oral Cholera Vaccines. Seoul, South Korea; 2012.
23. Mogasale V, Levin A, Maskery B, DeRoeck D, Kim YE, Clemens J, et al. Oral cholera vaccines to control endemic disease: an economic and epidemiological modelling analysis. Lancet. 2013;382(6).
24. Barendregt JJ. Ersatz. 1.1 ed. Brisbane: Epigear International (www.epigear.com); 2010.
25. WHO. The Global Task Force on Cholera Control 2019 [cited 2019 6, October]. Available from: https://www.who.int/cholera/task_force/en/.
26. Mogasale V, Ramani E, Wee H, Kim J. Oral Cholera Vaccination Delivery Cost in Low- and Middle-Income Countries: An Analysis Based on Systematic Review. PLoS Negl Trop Dis. 2016;10(12).
27. Hsiao A, Desai SN, Mogasale V, Exclerc J-L, Digilio L. Lessons learnt from 12 oral cholera vaccine campaigns in resource-poor settings. WHO Bull 2017;95:303–12.

For peer review only - http://bmjopen.bmj.com/site/about/guidelines.xhtml

Tables

Table 1: Input parameter assumptions used in uncertainty analysis

Input parameter	Mean value	Minimum value	Maximum value	Source
Out-of-pocket costs to patient and family for hospitalization	$38.87	$6.05	$109.94	(12-16)
Out-of-pocket costs to patient and family for outpatient cases	$5.71	$0.89	$16.15	(14)
Number of days with the loss of income — cholera cases	5.76 days	4.61 days	6.97 days	(12-16)
Number of days with the loss of income — caregivers	3.86 days	3.09 days	4.64 days	(12, 14, 15)
Public health service delivery costs for hospitalized cases	$65.77	$39.83	$85.41	(12-14)
Public health service delivery costs for outpatient cases	$2.84	$2.55	$3.31	(14)
Age of death due to cholera (years)	23.74	1.00	66.00	(20)
Proportion of cases hospitalized				
Base case	0.90	NA	NA	(12, 14, 15)
Scenario 1	0.75	NA	NA	(22, 23)
Scenario 2	0.55	NA	NA	(6)

Table 2: Economic burden of cholera in Africa

Economic burden	US$ 2015 (in millions)			I$ 2015 (in millions)		
	Mean	95%LCI	95%UCI	Mean	95%LCI	95%UCI
Lost productivity due to illness	$30.7	$22.4	$ 39.1	$72.8	$53.7	$ 92.1
Public health system costs	$58.9	$ 46.4	$70.8	$145.0	$114.8	$173.4
Out-of-pocket costs	$40.1	$16.2	$69.6	$97.6	$40.0	$168.4
Total economic burden excluding deaths	$129.6	$100.3	$164.2	$315.3	$245.0	$397.8
Lost productivity due to premature deaths	$876.2	$293.6	$1,174.8	$2,091.1	$721.7	$2,793.0
Total economic burden including deaths	$1,005.8	$426.4	$1,304.1	$2,406.4	$1,043.9	$3,107.5

For peer review only - http://bmjopen.bmj.com/site/about/guidelines.xhtml

Table 3: Economic burden of cholera in Africa under various cholera incidence scenarios

	US$ 2015				I$ 2015			
	Base estimate	Sensitivity 1	Sensitivity 2	WHO Report	Base estimate	Sensitivity 1	Sensitivity 2	WHO Report
Cases	1,008,642	1,756,703	504,321	71,176	1,008,642	1,756,703	504,321	71,176
Deaths	38,104	66,416	19,052	937	38,104	66,416	19,052	937
Productivity loss due to illness (millions)	$30.7	$53.9	$15.2	$1.7	$72.8	$131.8	$36.1	$4.0
Public health system costs(millions)	$58.9	$103.0	$29.4	$4.1	$145.0	$260.0	$72.5	$9.5
Out of pocket costs (millions)	$40.1	$70.3	$20.0	$2.8	$97.6	$176.2	$49.6	$6.2
Total economic burden excluding deaths (millions)	$129.6	$227.2	$64.6	$8.6	$315.3	$568.0	$157.2	$19.7
Productivity loss due to premature deaths (millions)	$876.2	$1,600.1	$454.7	$20.8	$2091.1	$3,925.1	$1,084.6	$48.1
Total economic burden including deaths (millions)	$1,005.8	$1,827.3	$519.3	$29.4	$2406.4	$4,493.1	$1,241.8	$67.8

WHO= World Health Organization

Table 4: Economic burden of cholera in Africa under various hospitalization and GDP per capita scenarios

	Bases case	Hospitalization rate 1*	Hospitalization rate 2*	GDP 1#	GDP 2#	GDP 3#
Cases	1,008,642	1,008,642	1,008,642	1,008,642	1,008,642	1,008,642
Deaths	38,104	38,104	38,104	38,104	38,104	38,104
Hospitalization rate (%)	90%	75%	55%	90%	90%	90%
Value of workdays as percentage of GDP per capita per day	100%	100%	100%	75%	50%	25%
Productivity loss due to illness (millions USD)	$30.5	$30.5	$30.5	$22.9	$15.3	$7.6
Public health system costs (millions USD)	$58.9	$49.9	$38.0	$58.9	$58.9	$58.9
Out of pocket costs (millions USD)	$39.9	$34.7	$27.7	$39.9	$39.9	$39.9
Sub-total economic burden (millions USD)	$129.2	$115.1	$96.2	$121.6	$114.0	$106.3
Productivity loss due to premature deaths (millions USD)	$909.3	$909.3	$909.3	$682.0	$454.7	$227.3
Total economic burden (millions USD)	$1,038.6	$1,024.4	$1,005.6	$803.6	$568.6	$333.7

* Percentage of cholera cases hospitalized is varied under this scenario

The workdays lost are valued at a certain percentage of gross domestic product (GDP) per capita per day

For peer review only - http://bmjopen.bmj.com/site/about/guidelines.xhtml

Figures

Figure 1: Spearman's rank correlation coefficient: Economic burden of cholera in Africa

For peer review only

For peer review only - http://bmjopen.bmj.com/site/about/guidelines.xhtml

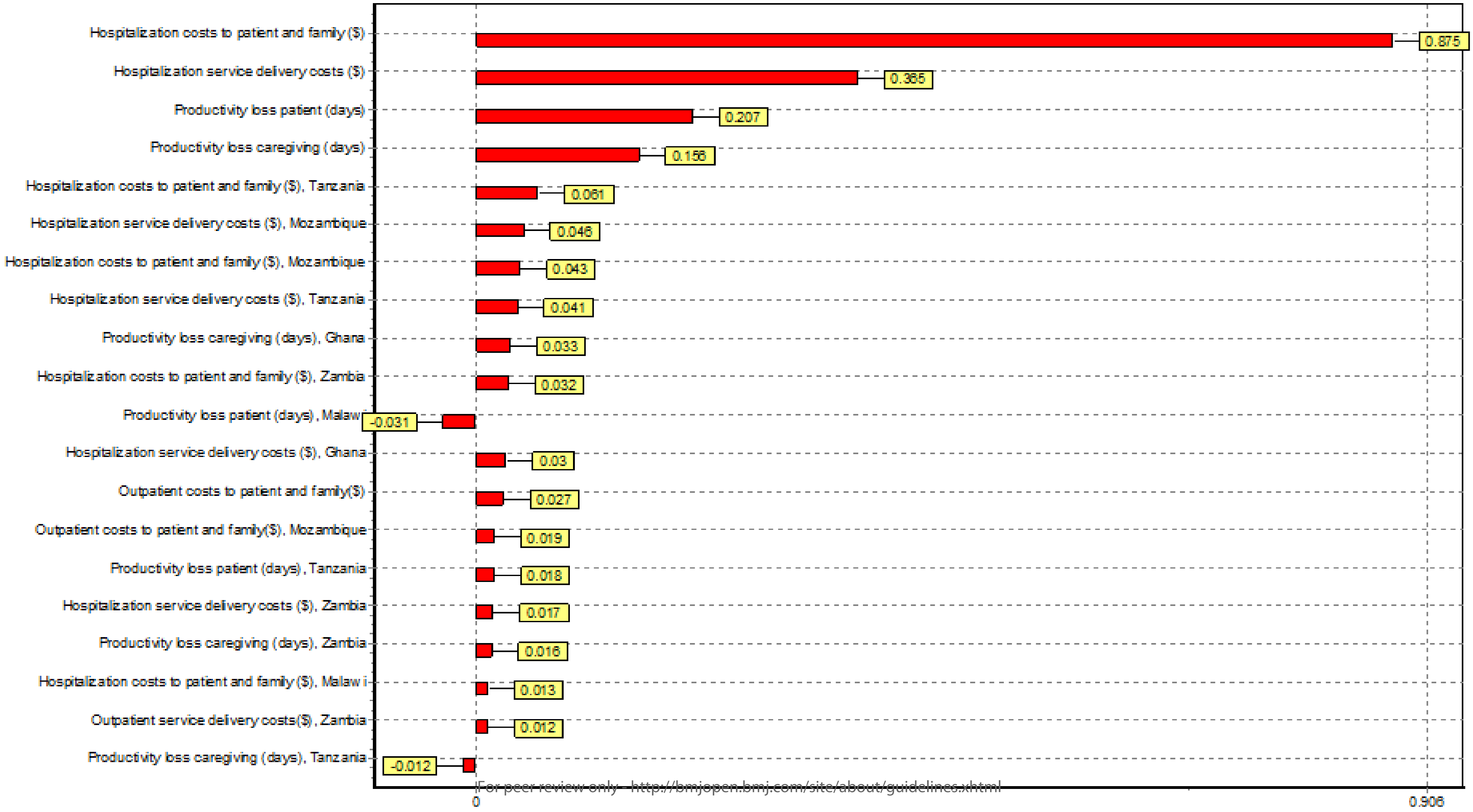

For peer review only - http://bmjopen.bmj.com/site/about/guidelines.xhtml

Annex

Annex1: *Comparison of methodology and results of Kirigia et al (5) and current paper*

Parameter	Kirigia et al (5)	Current paper
Year of analysis (disease burden)	2006, 2007, 2008	2015
Year of price	2002	2015
Data source for cholera cases	WHO reported	Global burden estimate and WHO reported
Household costs	Average (non-cholera) health care costs reported in WHO survey in 16 countries	Average cholera specific household costs reported in 5 cost of illness studies
Health system costs	The hotel were assumptions based on non-cholera cases reported by WHO. The medicines and diagnostic costs were estimated based on standard practices using assumptions.	Average of reported health system costs from 5 cost of illness studies for hospitalized cases and from WHO database of outpatients. Diagnostic costs are not included.
Indirect costs	Productivity loss for the duration of illness and premature deaths were valued by gross national income per capita	Productivity loss for the duration of illness and premature deaths were valued by gross domestic product per capita
Scenario analysis	For the year (2006, 2007, 2008), Life expectancy (73, 53, 40 years)	Disease burden (3 estimates and WHO reported), Hospitalization rate (90%, 75%, 55%), GDP per capita (100%, 75%, 50%, 25%)
Range of estimated economic burden in $2015 including mortality costs	$61.4 million to $245.5 million ($39 million and $156 million in 2002 prices)	$519.3 million to $1.8 billion based on estimated cases and $29.4 million based on WHO reported cases

(5) Kirigia JM, Sambo LG, Yokouide A, Soumbey-Alley E, Muthuri LK, Kirigia DG. Economic burden of cholera in the WHO African region. BMC international health and human rights. 2009;9:8.

For peer review only

Annex 2: Economic burden of cholera in Africa by country

Country*	Population in 2015 (2)	GDP per capita US$ 2015 (18)	GDP per capita I$ 2015 (18)	Infant mortality rate per 1000 live births (28)	Life expectancy at birth 2015(21)	Current health expenditure per capita (in 2015) (29)	Cases in 2015(2)	Deaths in 2015(2)	Productivity loss due to illness (US$2015)	Public health system cost (US$2015)	Out of pocket costs (US$2015)	Economic burden excluding deaths (US$2015)	Average cost of illness (US$2015)	Productivity loss due to premature deaths (US$2015)	Total economic burden (US$2015)	Cholera economic burden per capita in US$ 2015
Angola	27,884,380	$4,355	$7,412	57.8	61.2	$108.68	19,158	728	$2,225,925	$1,132,539	$792,875	$4,151,339	$216.69	$69,296,429	$73,447,768	$2.63
Benin	10,575,962	$1,077	$2,889	64.4	60.6	$ 31.29	4,755	181	$136,614	$277,839	$196,785	$611,239	$128.55	$4,209,382	$4,820,620	$0.46
Burkina Faso	18,110,616	$576	$1,704	53.6	59.9	$33.51	6,527	248	$100,233	$381,155	$270,122	$751,509	$115.14	$3,049,953	$3,801,463	$0.21
Burundi	10,160,034	$294	$769	45.8	57.1	$14.39	10,341	393	$81,249	$602,779	$427,970	$1,111,998	$107.53	$2,341,441	$3,453,440	$0.34
Cameroon	23,298,376	$1,339	$3,469	55.7	57.6	$63.63	9,487	361	$338,811	$555,189	$392,627	$1,286,627	$135.62	$9,873,360	$11,159,987	$0.48
Cape Verde	524,740	$2,997	$6,313	18.3	72.6	$159.22	117	4	$9,356	$6,900	$4,843	$21,099	$180.30	$338,692	$359,792	$0.69
Central Africa Rep.	4,493,171	$354	$698	90.7	51.4	$21.98	5,742	218	$54,200	$335,138	$237,633	$626,971	$109.19	$1,362,506	$1,989,477	$0.44
Chad	14,110,971	$947	$2,648	76.5	52.6	$35.52	11,486	436	$290,170	$671,161	$475,372	$1,436,704	$125.08	$7,527,860	$8,964,564	$0.64
Comoros	777,435	$1,260	$2,637	56	63.5	$58.24	68	3	$2,296	$3,992	$2,827	$9,115	$133.44	$74,080	$83,195	$0.11
Congo, Democratic Republic	76,244,532	$436	$760	73.9	59.2	$20.11	136,821	5,199	$1,591,458	$7,976,946	$5,662,446	$15,230,850	$111.32	$47,788,731	$63,019,580	$0.83
Congo, Rep.	4,856,093	$2,067	$7,318	38.9	64.1	$58.45	4,770	181	$263,037	$280,720	$197,393	$741,150	$155.39	$8,569,186	$9,310,337	$1.92
Cote d'voire	23,226,148	$1,397	$3,427	64.8	53.1	$75.45	15,181	577	$565,934	$888,232	$628,278	$2,082,444	$137.17	$14,868,449	$16,950,893	$0.73
Djibouti	913,998	$2,533	$4,611	54.2	62.3	$81.73	164	5	$11,091	$9,603	$6,793	$27,487	$167.46	$295,674	$323,160	$0.35
Eritrea	3,342,818	$352	$950	34.1	64.6	$27.18	12,860	489	$120,883	$750,166	$532,229	$1,403,279	$109.12	$3,968,339	$5,371,618	$1.61
Ethiopia	100,835,453	$703	$1,811	43.8	65.0	$25.11	195,093	7,414	$3,657,892	$11,379,858	$8,074,096	$23,111,846	$118.47	$120,778,513	$143,890,360	$1.43
Gabon	1,947,690	$7,453	$17,970	36.3	65.7	$197.94	405	15	$80,456	$24,265	$16,745	$121,467	$300.20	$2,680,703	$2,802,169	$1.44
Gambia, The	2,085,860	$650	$2,375	41.9	61.0	$21.85	370	14	$6,407	$21,581	$15,302	$43,290	$117.08	$198,500	$241,790	$0.12
Ghana	27,849,203	$1,754	$5,524	38.9	62.4	$82.41	6,794	258	$383,782	$439,711	$524,804	$1,348,298	$198.45	$10,087,937	$11,436,235	$0.41
Guinea	11,432,096	$712	$1,838	68.5	59.4	$42.25	5,656	215	$107,438	$329,968	$234,059	$671,465	$118.73	$3,239,028	$3,910,493	$0.34
Guniea-Bissau	1,737,207	$644	$1,679	59.5	57.0	$ 38.14	1,142	43	$19,619	$66,610	$47,263	$133,492	$116.89	$559,084	$692,575	$0.40
Kenya	47,878,339	$1,453	$3,235	33.3	66.7	$66.02	67,091	2,549	$2,601,329	$3,920,223	$2,776,614	$9,298,166	$138.59	$87,849,671	$97,147,837	$2.03

For peer review only

Lesotho	2,059,011	$1,167	$3,174	71.2	53.7	$97.47	1,768	67	$55,025	$103,310	$73,170	$231,505	$130.94	$1,466,453	$1,697,959	$0.82
Liberia	4,472,229	$764	$1,421	58.5	62.0	$71.94	2,137	81	$43,582	$124,613	$88,441	$256,636	$120.09	$1,370,686	$1,627,323	$0.36
Madagascar	24,234,080	$400	$1,470	40.8	65.5	$22.13	22,766	865	$243,165	$1,328,860	$942,189	$2,514,214	$110.44	$8,084,510	$10,598,724	$0.44
Malawi	16,745,305	$354	$1,129	40.3	62.7	$34.00	10,209	388	$118,651	$718,229	$267,074	$1,103,954	$108.14	$3,068,258	$4,172,211	$0.25
Mali	17,438,772	$773	$2,115	67.2	57.5	$30.83	10,070	383	$207,579	$588,249	$416,755	$1,212,582	$120.42	$6,033,505	$7,246,088	$0.42
Mauritania	4,046,304	$1,303	$4,297	55.4	63.1	$53.61	3,609	137	$125,505	$211,067	$149,361	$485,933	$134.64	$4,021,206	$4,507,139	$1.11
Mozambique	27,042,001	$529	$1,195	59.3	57.7	$27.38	50,811	1,931	$655,324	$2,409,795	$1,875,380	$4,940,499	$97.23	$20,923,973	$25,864,472	$0.96
Namibia	2,314,901	$5,160	$11,320	33.5	63.8	$466.11	610	23	$83,980	$36,017	$25,245	$145,241	$238.10	$2,701,262	$2,846,503	$1.23
Niger	20,001,663	$363	$964	52	59.7	$25.72	16,212	616	$156,885	$945,879	$670,946	$1,773,710	$109.41	$4,750,822	$6,524,533	$0.33
Nigeria	181,137,454	$2,726	$6,053	78.7	53.0	$97.52	134,155	5,098	$9,758,067	$7,856,671	$5,552,111	$23,166,849	$172.69	$255,879,611	$279,046,459	$1.54
Rwanda	11,369,066	$736	$1,907	30.8	66.7	$46.40	15,171	577	$298,052	$885,490	$627,864	$1,811,406	$119.40	$10,076,243	$11,887,649	$1.05
São Tomé and Principe	199,439	$1,570	$3,033	27	66.5	$86.91	39	1	$1,633	$2,282	$1,614	$5,529	$141.78	$37,118	$42,647	$0.21
Senegal	14,578,450	$1,187	$3,118	35.1	66.8	$50.96	7,252	276	$229,646	$424,220	$300,130	$953,996	$131.55	$7,777,910	$8,731,906	$0.60
Sierra Leone	7,171,909	$600	$1,433	87.8	51.4	$119.82	5,177	197	$82,813	$302,022	$214,254	$599,089	$115.72	$2,085,448	$2,684,538	$0.37
Somalia	13,797,204	$293	$775	83.1	55.9	NA	11,220	359	$87,847	$656,748	$464,349	$1,208,944	$107.75	$2,076,914	$3,285,858	$0.24
South Sudan	10,715,657	$1,225	$2,002	63.7	56.3	$28.05	17,098	650	$558,887	$1,000,988	$707,614	$2,267,490	$132.62	$15,851,280	$18,118,769	$1.69
Sudan	38,902,948	$1,698	$4,282	45.4	64.3	$158.02	26,311	842	$1,191,987	$1,540,819	$1,088,902	$3,821,708	$145.25	$32,772,322	$36,594,030	$0.94
Swaziland	1,104,038	$3,780	$10,038	47.9	57.1	$231.42	1,384	53	$139,559	$81,285	$57,278	$278,122	$200.96	$4,053,661	$4,331,783	$3.92
Tanzania	51,482,638	$948	$2,782	41.9	65.0	$32.09	84,550	3,213	$1,945,796	$5,254,900	$2,174,112	$9,374,807	$110.88	$70,515,725	$79,890,533	$1.55
Togo	7,323,162	$564	$1,546	51.4	59.9	$35.21	4,919	187	$73,986	$287,293	$203,577	$564,855	$114.83	$2,253,130	$2,817,985	$0.38
Uganda	38,225,447	$647	$2,272	38.6	59.6	$39.77	38,066	1,446	$656,973	$2,222,025	$1,575,392	$4,454,389	$117.02	$19,855,976	$24,310,365	$0.64
Zambia	15,879,370	$1,310	$3,844	44.5	61.4	$58.52	20,618	784	$749,934	$1,203,369	$440,605	$2,393,908	$116.11	$22,512,900	$24,906,808	$1.57
Zimbabwe	13,814,642	$1,425	$2,640	38.5	60.4	$94.29	10,462	398	$397,751	$612,289	$432,978	$1,443,018	$137.93	$12,217,700	$13,660,718	$0.99
Total/average	936,340,81			52.26	60.61		1,008,642	38,104	$30,510,80	$58,850,99	$39,866,41	$129,228,2		$909,344,1	$1,038,572,	$39.20

For peer review only

	2								7	3	8	18		33	351	

For peer review only
