# Supplementary material for: Model-based estimation of the economic burden of cholera in Africa
Source: BMJ Open. 2021 Mar 23;11(3):e044615. doi: 10.1136/bmjopen-2020-044615 (PMC7993295; doi:10.1136/bmjopen-2020-044615)
Supplement: Reviewer comments [file bmjopen-2020-044615.reviewer_comments.pdf]

PEER REVIEW HISTORY

BMJ Open publishes all reviews undertaken for accepted manuscripts. Reviewers are asked to complete a checklist review form (**http://bmjopen.bmj.com/site/about/resources/checklist.pdf**) and are provided with free text boxes to elaborate on their assessment. These free text comments are reproduced below.

ARTICLE DETAILS

**TITLE (PROVISIONAL)**	Model-based estimation of the economic burden of Cholera in Africa
**AUTHORS**	Mogasale, Vittal; Ngogoyo, Samuel; Mogasale, Vijayalaxmi

VERSION 1 – REVIEW

**REVIEWER**	Mercy Mvundura<br>PATH USA
**REVIEW RETURNED**	09-Dec-2020

**GENERAL COMMENTS**	The authors have adequately addressed the comments I provided.

**REVIEWER**	Dr Belinda Archibong<br>Barnard College, USA
**REVIEW RETURNED**	20-Dec-2020

**GENERAL COMMENTS**	Minor grammatical errors (spelling of IMF), so be careful of that. Otherwise, the author has adequately addressed the previous review concerns.

VERSION 1 – AUTHOR RESPONSE

Reviewer: 1
Comments to the Author:
The authors have adequately addressed the comments I provided.

Reviewer: 2
Minor grammatical errors (spelling of IMF), so be careful of that. Otherwise, the author has adequately addressed the previous review concerns.

Response: Spelling errors of IMF is corrected. Checked for other grammatical errors. Minor edits are done.
